# Structure determination using high-order spatial correlations in single-particle X-ray scattering

**DOI:** 10.1107/S2052252523009831

**Published:** 2024-01-01

**Authors:** Wenyang Zhao, Osamu Miyashita, Miki Nakano, Florence Tama

**Affiliations:** a Computational Structural Biology Research Team, RIKEN Center for Computational Science, 6-7-1 Minatojima-minamimachi, Chuo-ku, Kobe, Hyogo 650-0047, Japan; b Institute of Transformative Bio-Molecules, Nagoya University, Furo-cho, Chikusa-ku, Nagoya, Aichi 464-8601, Japan; c Department of Physics, Graduate School of Science, Nagoya University, Furo-cho, Chikusa-ku, Nagoya, Aichi 464-8601, Japan; Uppsala University, Sweden

**Keywords:** 3D reconstructions, single-particle imaging, X-ray free-electron lasers, spatial correlations, structure determination, single-particle X-ray scattering, diffraction patterns, detector noise

## Abstract

Single-particle imaging using X-ray free-electron lasers (XFELs) is a promising technique for observing nanoscale biological samples under near-physiological conditions. However, as the sample’s orientation in each diffraction pattern is unknown, advanced algorithms are required to reconstruct the 3D diffraction intensity volume and subsequently the sample’s density model. While most approaches perform 3D reconstruction via determining the orientation of each diffraction pattern, a correlation-based approach utilizes the averaged spatial correlations of diffraction intensities over all patterns, making it well suited for processing experimental data with a poor signal-to-noise ratio of individual patterns. Here, a method is proposed to determine the 3D structure of a sample by analyzing the double, triple and quadruple spatial correlations in diffraction patterns. This *ab initio* method can reconstruct the basic shape of an irregular unsymmetric 3D sample without requiring any prior knowledge of the sample. The impact of background and noise on correlations is investigated and corrected to ensure the success of reconstruction under simulated experimental conditions. Additionally, the feasibility of using the correlation-based approach to process incomplete partial diffraction patterns is demonstrated. The proposed method is a variable addition to existing algorithms for 3D reconstruction and will further promote the development and adoption of XFEL single-particle imaging techniques.

## Abstract 2

A method is described for determining the 3D structure of a sample from X-ray free-electron laser single-particle diffraction patterns by analyzing different orders of spatial correlations of diffraction intensities.

## 1. Introduction

Human beings have never stopped exploring the microscopic universe of microorganisms, cells, organelles and biomacromolecules. Over the past four centuries, advances in microscopy have been significant, with updated probes including visible light, electrons and X-rays. In the past two decades, coherent X-ray diffraction imaging (CXDI) (Miao *et al.*, 1999 ▸) has emerged as an innovative microscopy technique. It uses coherent X-rays and records diffraction patterns to recover a sample’s density distribution. Unlike other imaging techniques, the resolution of CXDI is not limited by the aberration of lenses, and hence its theoretical resolution can reach the level of X-ray wavelength, of the order of Ångstroms. On this basis, single-particle imaging using extremely strong pulses from X-ray free-electron lasers (XFELs) appears particularly appealing as it can avoid radiation damage resulting from prolonged exposure. The technique involves continuously feeding identical copies of a sample into the laser focus spot and capturing a diffraction snapshot before each copy is destroyed by the intense laser pulses (Neutze *et al.*, 2000 ▸). By collecting and analyzing a set of diffraction patterns, it is possible to reconstruct the 3D diffraction intensity volume of the sample and subsequently recover the sample’s 3D density model. Since this technique can measure biological samples under near-physiological conditions without being crystallized or cryogenic, it is considered as a promising complement to X-ray crystallography and cryo-electron microscopy. In the past decade, along with the construction of XFEL facilities and the development of instruments including detectors and sample-delivery systems, a number of successful demonstrations on nanomaterials (Xu *et al.*, 2014 ▸; Nakano *et al.*, 2022 ▸), single cells (Kimura *et al.*, 2014 ▸; van der Schot *et al.*, 2015 ▸), organelles (Hantke *et al.*, 2014 ▸; Takayama *et al.*, 2015 ▸), viruses (Seibert *et al.*, 2011 ▸; Ekeberg *et al.*, 2015 ▸; Hosseinizadeh *et al.*, 2017 ▸) and biocomplexes (Gallagher-Jones *et al.*, 2014 ▸) have been reported.

In addition to the instruments, the development of advanced algorithms is also crucial for XFEL single-particle imaging. As the sample’s orientation in each diffraction pattern is unknown, reconstructing the 3D diffraction intensity volume without this critical information poses a significant analytical challenge. So far, several classes of approaches have been developed. The common arc approach (Huldt *et al.*, 2003 ▸; Shneerson *et al.*, 2008 ▸; Bortel & Tegze, 2011 ▸; Yefanov & Vartanyants, 2013 ▸) identifies the common intersection curve in each pair of patterns and then determines the relative orientations of all patterns. The correlation maximization approach (Tegze & Bortel, 2012 ▸, 2021 ▸; Nakano *et al.*, 2018 ▸), as well as the expansion maximization compression method (Loh & Elser, 2009 ▸), iteratively optimizes a tentative 3D diffraction intensity volume by inserting back each pattern at its most probable orientation in that volume. The manifold embedding approach (Fung *et al.*, 2009 ▸; Giannakis *et al.*, 2012 ▸; Schwander *et al.*, 2014 ▸) estimates the orientations of all patterns by mapping a high-dimensional space of diffraction patterns, known as the manifest space, to a 3D orientation space, known as the manifold. Despite their differing details, we collectively refer to these approaches as orientation-based approaches since they all aim to find the orientation or the probability distribution of the orientation of each diffraction pattern.

In this work, we focus on a distinct approach: the correlation-based approach. Unlike the orientation-based approaches mentioned above, it does not assign the orientation of each diffraction pattern. Instead, it computes spatial correlations of diffraction intensities in all patterns. Assuming that all patterns are randomly oriented, which is typically the case in XFEL single-particle imaging, it is possible to reconstruct the 3D diffraction intensity volume directly by analyzing the correlations. The correlation-based approach was initially proposed by Kam (1977 ▸, 1980 ▸) for processing electron micrographs more than 40 years ago and regained much attention 30 years later in the field of XFELs (Saldin, Shneerson *et al.*, 2010 ▸; Saldin, Poon *et al.*, 2010 ▸; Saldin *et al.*, 2011 ▸; Kirian, 2012 ▸). Very recently, it has had some new theoretical developments again in the field of cryo-electron microscopy (Lan *et al.*, 2022 ▸; Bendory, Khoo *et al.*, 2023 ▸; Bendory, Boumal *et al.*, 2023 ▸). As the correlation-based approach can accumulate signals by averaging the correlations over all patterns, it is well suited for processing experimental data with a poor signal-to-noise ratio (SNR) of individual patterns, which is a major challenge in current XFEL single-particle imaging. Furthermore, as noted in its first proposal, it can process experimental data of fluctuation X-ray scattering (Kam *et al.*, 1981 ▸; Pande *et al.*, 2018 ▸), which captures the diffraction pattern of multiple copies of the sample in one snapshot. For these reasons, the correlation-based approach warrants further attention and investigation.

However, reconstructing the 3D diffraction intensity volume from correlations is not trivial. In most reports, the complexity of the reconstruction is simplified by either restricting the rotation to only one axis (Pedrini *et al.*, 2013 ▸) or using cylindrically symmetric samples (Starodub *et al.*, 2012 ▸; Chen *et al.*, 2012 ▸). For 3D reconstruction of an irregular sample rotated in any direction in 3D space, Donatelli *et al.* developed a multitiered iterative phasing (MTIP) method (Donatelli *et al.*, 2015 ▸, 2017 ▸; Kommera *et al.*, 2021 ▸) to optimize the 3D diffraction intensity volume and retrieve its phases simultaneously. This algorithm has been successfully applied to experimental data of XFEL single-particle imaging (Kurta *et al.*, 2017 ▸) and fluctuation X-ray scattering (Pande *et al.*, 2018 ▸). Furthermore, von Ardenne *et al.* (2018 ▸) proposed algorithms to determine the 3D diffraction intensity volume by using three-photon correlations and Monte Carlo simulated annealing. Still, further discussion on the correlation-based approach is needed to improve its robustness and applicability in processing diverse experimental data.

In this work, we explore an alternative method using high-order correlations. Just as many algorithms in XFEL single-particle imaging are inspired by those developed for cryo-electron microscopy, the basic idea of this method was first described by Kam & Gafni (1985 ▸) for reconstructing the structure of human wart virus from electron micrographs. However, it has seldom been implemented since then. In the present work, for the first time, we complete this method with essential technical details, illuminating how it can be practically implemented through numerical procedures, and we also evaluate its performance in XFEL single-particle imaging. More importantly, we investigate the impact of various sources of noise and backgrounds on correlations, and present formulas to correct the impact. This innovation of noise correction is crucial for making the method practically workable, at least under simulated experimental conditions. Additionally, we demonstrate the feasibility of using the correlation-based approach to process incomplete partial diffraction patterns. We also discuss its differences from and potential connections to other approaches.

## 2. Theory

### 2.1. Reconstructing 3D diffraction intensity volume using double, triple and quadruple correlations

We express the 3D diffraction intensity volume in spherical coordinates as *I*(*k*, θ, ϕ). It can be expanded as a linear combination of spherical harmonics (SH) *Y*_*l*,*m*_(θ, ϕ) of degree *l* and order *m* as

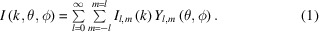

Thus, once the SH coefficients *I*_*l*,*m*_(*k*) are obtained from experimental diffraction patterns, the 3D diffraction intensity volume can be reconstructed.

A diffraction pattern recorded by a planar detector is a part of an Ewald sphere. We first resample the recorded pattern in polar coordinates as *I*(ρ, ϕ) by using bicubic interpolation, and then find its coordinates in reciprocal space as **k** = (*k*, θ, ϕ) via 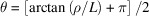 and 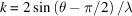 , where *L* is the sample-to-detector distance and λ is the incident wavelength. The double correlations are computed via

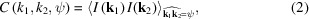

where the angle brackets denote the average, first over all available (**k**_1_, **k**_2_) pairs in the same pattern, with the included angle from **k**_1_ to **k**_2_ being ψ, and then over all patterns. The included angle ψ from **k**_1_ to **k**_2_ is calculated using the following equation:

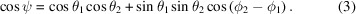

When the Ewald sphere is approximated as flat, it follows that θ(*k*_1_) = θ(*k*_2_) = π/2, and thus ψ = ϕ_2_ − ϕ_1_.

At each (*k*_1_,*k*_2_), we expand the double correlations 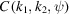 into Fourier–Legendre series as

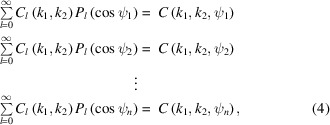

where *P*_*l*_ is the Legendre polynomial of degree *l* and *n* is the number of ψ angle points. In the practical applications, we set the upper limit of *l* as *l*_max_. Thus, in the linear system in equation (4), the number of unknown Fourier coefficients *C*_*l*_(*k*_1_,*k*_2_) is *l*_max_ + 1. In general, this number is much smaller than the number of sub-equations *n*. Therefore, we can obtain *C*_*l*_(*k*_1_,*k*_2_) for 0 ≤ *l* ≤ *l*_max_ by solving the overdetermined linear system (Aster *et al.*, 2013 ▸). We refer to the *k*_max_ × *k*_max_ square matrix **C**_*l*_ as the partial correlation matrix in the *l* subspace.

The partial correlation matrices **C**_*l*_ for 0 ≤ *l* ≤ *l*_max_ are obtained from experimental diffraction patterns. In parallel, they are theoretically related to the SH coefficients *I*_*l*,*m*_(*k*) of the 3D diffraction intensity volume (Kam, 1977 ▸) as

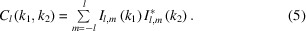

Here, we consider expressing *I*_*l*,*m*_(*k*) as a linear combination of basis vectors obtained from **C**_*l*_. To obtain the basis vectors, we decompose **C**_*l*_ into its normalized eigenvectors *u*_*l*,*i*_(*k*) and the corresponding eigenvalues λ_*l*,*i*_ for 1 ≤ *i* ≤ *k*_max_. Since the 3D diffraction intensity volume *I*(*k*, θ, ϕ) is real, we have 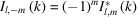 . Thus, **C**_*l*_ should be real and symmetric. All of its eigenvectors are orthogonal to each other and all of its eigenvalues are real. The number of non-zero eigenvalues equals the rank of **C**_*l*_. As **C**_*l*_ should also be a real Gram matrix, all of its eigenvalues are non-negative, and its rank is no larger than 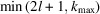 . Therefore, **C**_*l*_ has at most 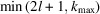 positive eigenvalues. We define the *i*th basis vector in the *l* subspace as *c*_*l*,*i*_ *u*_*l*,*i*_(*k*), where *c*_*l*,*i*_ = (λ_*l*,*i*_)^1/2^ and *i* ranges from 1 to 2*l* + 1. Note that *c*_*l*,*i*_ is zero when *i* > *k*_max_.

Using these basis vectors, the SH coefficients can be expressed as

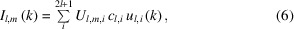

where *U*_*l*,*m*,*i*_ are the elements of a (2*l* + 1) × (2*l* + 1) unitary matrix **U**_*l*_, as a requirement to satisfy equation (5). Since **U**_*l*_ can be an arbitrary unitary matrix, each basis vector can be freely aligned, and correspondingly the SH coefficients cannot be fixed. That is to say, when considering only correlations *C*, the 3D diffraction intensity volume has a general solution and an infinite number of possibilities. Additional information is needed for 3D reconstruction.

In this work, we compute high-order correlations and use the constraint between different orders of correlations to narrow down the general solution to particular solutions. Quadruple correlations *D*(*k*_1_,*k*_2_,ψ) are computed to characterize the 3D squared diffraction intensity volume *S*(*k*, θ, ϕ):

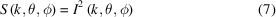

and

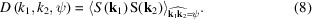

In analogy to equations (4)–(6) [Disp-formula fd6], the SH coefficients *S*_*l*,*m*_(*k*) of *S*(*k*, θ, ϕ) can again be expressed as a linear combination of the basis vectors *d*_*l*, *j*_ *v*_*l*, *j*_(*k*) obtained from partial correlation matrices **D**_*l*_, as 

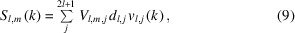

where *V*_*l*,*m*, *j*_ are the elements of a unitary matrix **V**_*l*_.

In the reconstruction, the 3D diffraction intensity volume to be determined must satisfy equations (6) and (9) simultaneously. This constraint determines the particular solutions of the SH coefficients and thus the volume. However, searching for the answer in the **U**_*l*_ and **V**_*l*_ sets for 0 ≤ *l* ≤ *l*_max_, which are indirectly coupled through equation (7), remains a challenging task.

To find the answer for the coupled **U**_*l*_ and **V**_*l*_ sets, we first assume a set of unitary matrices 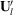 for 0 ≤ *l* ≤ *l*_max_. Then, we calculate the corresponding 3D diffraction intensity volume *I*′(*k*, θ, ϕ) via equations (6) and (1). After that, the corresponding 3D squared diffraction intensity volume *S*′(*k*, θ, ϕ) is expanded into SH coefficients 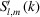 . The corresponding set of matrices 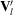 are calculated by inverting equation (9), taking advantage of the fact that the eigenvectors *v*_*l*, *j*_(*k*) within the same *l* subspace are orthogonal to each other:

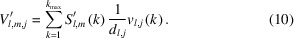

In equation (10), *d*_*l*, *j*_ and *v*_*l*, *j*_(*k*) are computed from experiment diffraction patterns, whereas the SH coefficients 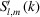 are expanded from the square of the assumed 3D diffraction intensity volume. For an arbitrary assumed set of unitary matrices 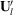 and the corresponding assumed 3D volume, the resulting matrices 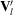 are not guaranteed to be unitary.

In order to search for the set of 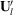 that makes all 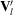 unitary, we need to define an objective function that is numerically optimizable. For this purpose, the introduction of triple correlations is essential. We define a constraint matrix 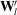 to summarize the coupling relation between 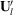 and 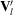 . This matrix 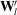 has dimensions of (2*l* + 1) × (2*l* + 1). Its (*j*, *i*) element is calculated via 

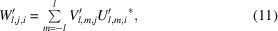

where the star superscript denotes conjugation.

The equivalent constraint matrices **W**_*l*_ can be estimated from experimental data. For this, we compute triple correlations *T* from all experimental diffraction patterns:

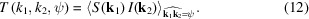

Again, partial correlation matrices **T**_*l*_ for 0 ≤ *l* ≤ *l*_max_ are obtained by solving a linear system similar to that in equation (4). Using a relation analogous to equation (5), and equations (6) and (9), the (*j*, *i*) element in the experimental constraint matrix **W**_*l*_ is calculated via

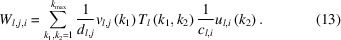

We need to ensure that the assumed constraint matrices 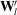 are equal to the matrices **W**_*l*_ that are directly obtained from the experimental diffraction patterns. If the assumed constraint matrices 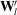 are equal to the experimental constraint matrices **W**_*l*_ for all degrees of *l* from 0 to *l*_max_, the assumed 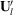 sets and the corresponding 3D diffraction intensity volume are valid. In this way, the process of searching for the answer can be simplified into minimizing a non-linear error function 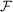 :

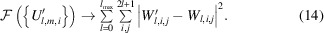




### 2.2. Implementation of the algorithm

While most of the formulas in Section 2.1 were first described by Kam in 1985 (Kam & Gafni, 1985 ▸), some additional technical details are crucial to making the reconstruction method work effectively. In this section, we will present the necessary supplementary formulas and illustrate how this reconstruction method can be practically implemented. We will also point out some factors essential for the rationality and success of this method.

The primary concern is to construct the unitary matrices 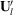 , which serve as the input of the error function 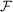 in equation (14). A commonly used method to construct a unitary matrix is QR decomposition of a complex square matrix. However, a unitary matrix of dimension *N* has *N* ^2^ degrees of freedom, whereas a complex square matrix has 2*N* ^2^ variables. Obviously, the constructed unitary matrix and the set of 2*N* ^2^ variables are not one-to-one mapped. This can fail the numerical optimization of error function 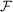 . Another well known method is to set *N* ^2^ independent variables of Euler angles, as once described by Zyczkowski & Kus (1994 ▸) and referred to as Hurwitz’s method here. However, it is unsuitable for the present work for reasons explained later. In the end, we formulated our own method to construct unitary matrices, as described below.

In a specified *l* subspace, the unitary matrix 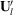 has a dimension *N* = 2*l* + 1. When *l* = 0, we set 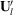 to be either [+1] or [−1]. When *l* > 0, 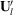 has *N* ^2^ degrees of freedom, and therefore its reconstruction requires *N* ^2^ independent variables. However, in this work, we restrict its freedom to *N*(*N* − 1)/2 to ensure the symmetry of 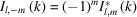 and to ensure that the corresponding 3D diffraction intensity volume *I*(*k*, θ, ϕ) is always real. We assume that all variables {α_*i*_} are angle values, where α_*i*_ ∈ [0, 2π) for 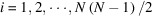 .

First, we use the first (*N* − 1) variables to construct a unit vector in an *N*-dimensional unit sphere:

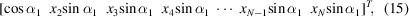

where

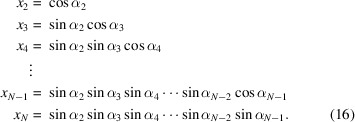

Second, we construct other (*N* − 1) *N*-dimensional unit vectors that are orthogonal to the initial unit vector and to each other. The resulting vectors form an orthogonal matrix **O**_*N*_ (Mayer, 2003 ▸):

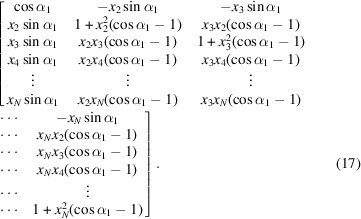

In a similar way, we use the next (*N* − 2) variables to construct an orthogonal matrix **O**_*N*−1_. This process is repeated until **O**_*N*−2_, …, **O**_2_ are constructed using all remaining α_*i*_ angles.

Third, we transform the right-most two column vectors in **O**_3_ by multiplying them by **O**_2_ from right, and recursively repeat this process until the right most (*N* − 1) columns in **O**_*N*_ have been transformed. The generated matrix **R**_*N*_ is a special orthogonal matrix with *N*(*N* − 1)/2 degrees of freedom.

Finally, we convert **R**_*N*_ to 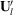 . Assuming the elements in the matrix 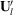 are indexed with (*m*, *i*) where −*l* ≤ *m* ≤ *l* and 1 ≤ *i* ≤ 2*l* + 1, the conversion is given by

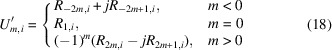

where *j* denotes an imaginary number.

In the reconstruction, we do not need to align all basis vectors (in other words, determine all column vectors) in the corresponding unitary matrices 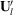 . Similar to singular value decomposition, the most significant features of an object are represented by the top few principal components. In this work, the 3D diffraction intensity volume *I*(*k*, θ, ϕ) is constructed by aligning basis vectors obtained through eigen decomposition of partial correlation matrices **C**_*l*_ for 0 ≤ *l* ≤ *l*_max_. The major features of *I*(*k*, θ, ϕ) can be sufficiently captured by aligning only a few basis vectors with large norms, rather than aligning all basis vectors. In each *l* subspace, it is possible to limit the number of employed basis vectors to a certain *i*_max,*l*_ in equation (6) and *j*_max,*l*_ in equation (9). Reducing the number of employed basis vectors can reduce the number of independent and dependent variables in the error function 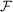 in equation (14). This will greatly simplify the complexity of the minimization process. To specify, if there are only *i*_max,*l*_ basis vectors employed in the *l* subspace, according to equation (6), we only need to construct the left-most *i*_max,*l*_ columns in 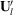 rather than the entire unitary matrix 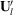 . In this case, only orthogonal matrices **O**_*N*_, **O**_*N*−1_, …, 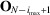 are necessary. The required number of independent variables is reduced to *i*_max_(2*l* + 1) − *i*_max_(*i*_max_ + 1)/2. As a comparison, Hurtwitz’s method to construct unitary matrices lacks this flexibility. In Hurtwitz’s method, no matter how many column vectors are required, *N* ^2^ independent variables are always needed.

The secondary concern is to introduce weight factors. This is to ensure the rationality of the error function 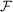 in equation (14). According to equations (6), (9) and (11), the (*j*, *i*) element in 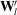 is the inner product of the *i*th unit column vector in 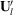 and the *j*th column vector in 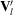 , which align the basis vectors *c*_*l*,*i*_ *u*_*l*,*i*_(*k*) and *d*_*l*, *j*_ *v*_*l*, *j*_(*k*), respectively. We need to account for the influence of the norms *c*_*l*,*i*_ and *d*_*l*, *j*_ on the 3D diffraction intensity volume *I*′(*k*, θ, ϕ) and 3D squared diffraction intensity volume *S*′(*k*, θ, ϕ), respectively. Therefore, we introduce a weighting factor of *c*_*l*,*i*_(*d*_*l*, *j*_)^1/2^ for *W*_*l*, *j*,*i*_ in the error function 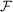 in equation (14) so that the basis vectors with larger norms are aligned more carefully than those with smaller norms.

The third concern is about the unitary check of 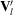 . In the 3D reconstruction, we aim to find an answer that allows both 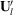 and 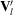 to be unitary. However, given an arbitrary set of unitary matrices 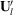 for 0 ≤ *l* ≤ *l*_max_, the unitarity of the corresponding 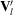 is not guaranteed. Our experience has shown that it is impractical to include the unitary check of 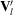 in the error function 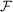 in equation (14), as the iterative optimization process can easily get trapped at the very beginning. However, we have noticed that checking the unit norm of the first column vector in 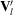 is essential when there is only one basis vector *d*_*l*,1_ *v*_*l*,1_(*k*) employed in the *l* subspace. The weighting factor of the check is *d*_*l*,*i*_. This unit-norm check determines the success or failure of reconstructing cylindrically symmetric models. We do not check 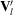 when there are more than one basis vectors employed in the *l* subspace.

In this work, the minimization of the error function 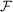 in equation (14) is achieved through progressive and iterative optimization. The iterative optimization is performed using the trust region reflective algorithm (Branch *et al.*, 1999 ▸), implemented by the least_squares function in the Python package *scipy.optimize*. Additionally, aligning all basis vectors simultaneously is a challenging task. To simplify, we begin by aligning the basis vectors with larger norms and then gradually introduce those with smaller norms. After finalizing each alignment, the optimized results become the initial parameters for the next alignment, in which the previously aligned basis vectors are realigned simultaneously with the newly introduced basis vector. This progressive approach ensures a robust process of optimization. In the *l* subspace, when a certain number of basis vectors *c*_*l*,*i*_ *u*_*l*,*i*_(*k*) are employed for constructing the 3D diffraction intensity volume via equation (6), we cannot accurately decide the optimum number of basis vectors *d*_*l*, *j*_ *v*_*l*, *j*_(*k*) that are employed for characterizing the corresponding 3D squared diffraction intensity volume via equation (9). As a compromise, we set these two numbers as equal.

### 2.3. Correcting the impact of noise and background on correlations

In practical experiments, the measured coherent diffraction patterns contain not only the coherent scattering signals from the sample but also scattering backgrounds from various sources. The measurement is also affected by multiple types of noise. For many years, it has been recognized that, compared with double correlations, high-order correlations are more susceptible to noise (Kirian, 2012 ▸; von Ardenne *et al.*, 2018 ▸; Singer, 2019 ▸). This is probably the reason why the reconstruction method in this work, although its basic idea was described nearly 40 years ago, has seldom been implemented. However, in this section, we will demonstrate that the impact of noise on high-order correlations can be eliminated as long as the second moment of the noise can be estimated. This noise elimination has not been reported before in the field of XFEL imaging; however, it is exactly the key that makes the present reconstruction method potentially applicable to noisy experimental data. We will also present the formulas to subtract the impact of background on correlations.

In our model under simulated experimental conditions, we use *I*(**k**) to denote the coherent scattering signal, *I*_Compt_(*k*) to denote the incoherent (Compton) scattering background from the sample itself and *I*_inst_(*k*) to denote the scattering backgrounds from the instruments, the environment or the solution used to preserve the sample, which are collectively referred to as the instrument background. The intensities of *I*(**k**), *I*_Compt_(*k*) and *I*_inst_(*k*) together form the overall source intensity *I*_s_(**k**) to be measured:

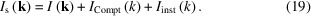

We further assume that the measurement of *I*_s_(**k**) is affected by photon shot noise, denoted as Δ_s_(**k**), and detector noise, including Fano noise and system electronic noise, denoted as Δ_d_(**k**). We use *I*_d_(**k**) to denote the intensity measured by the detector:

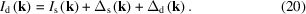

Detailed explanations on *I*_Compt_, *I*_inst_, Δ_s_ and Δ_d_ are presented in Appendix *A* [App appa].

We first eliminate the impact of photon shot noise and detector noise on the correlations, and then we discuss the process of background subtraction. The correlations *C*_d_, *T*_d_ and *D*_d_ are directly computed from the measured patterns *I*_d_(**k**). After noise elimination, the correlations *C*_s_, *T*_s_, and *D*_s_ characterizing the source intensity *I*_s_(**k**) can be obtained. According to the mean and the second moment of Δ_s_(**k**) and Δ_d_(**k**) in equations (A8), (A9), (A14) and (A15), when **k**_1_ ≠ **k**_2_, the equations for noise elimination are given as follows:

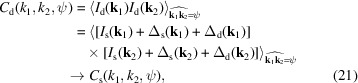




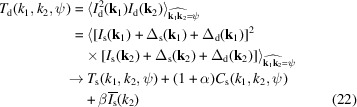

and

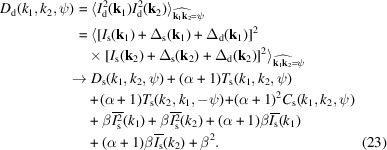

In equations (22) and (23), 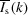 is the angular average of the source intensity *I*_s_(**k**). It can be obtained by averaging all the measured patterns *I*_d_(**k**):

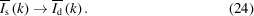




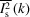
 is the angular average of the square of the source intensity *I*_s_(**k**). It can be obtained by averaging the square of all the measured patterns *I*_d_(**k**) and then making corrections:

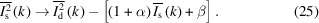

In equations (22), (23) and (25), α and β are parameters related to the detector’s Fano noise and system electronic noise, respectively. Their definitions and typical values are given in Appendix *A*4[App appa]. When their values are small enough, the correction terms containing α and β can be approximately omitted. Then, the above equations are solely concerned with eliminating the photon shot noise. In this work, we assume that the values of α and β can be accurately determined according to the detector’s specifications and remain constant. However, in a real experiment, they may fluctuate with changes in environmental conditions and the detector’s state. Nevertheless, we can approximately ignore their fluctuations and focus solely on the overall statistical distribution of detector noise, as explained in Section S6 of the supporting information. Furthermore, as indicated in Appendix *A*4[App appa], when using modern X-ray detectors, the values of α and β typically fall within the order of 10^−4^, suggesting that the impact of detector noise on high-order correlations is relatively minor. For this reason, if the values of α and β are unknown, we can attempt 3D reconstructions by initially setting them to zero and then gradually adjusting their values based on the quality of the reconstructions.

According to equation (21), noise elimination is not needed for the double correlations *C*_d_. This is because the mean of the noise is zero. On the other hand, the noise elimination for *T*_d_ and *D*_d_ in equations (22) and (23) relies on the second moment of the noise. When resampling the measured diffraction patterns by interpolation, the second moment of the noise may change, and the correction terms in equations (22) and (23) should also change accordingly. A simple approach is to use the nearest interpolation method. The roughness of correlation functions caused by nearest interpolation can be addressed separately using smoothing methods, when necessary.

Very recently, the idea of using the second moment of noise to obtain unbiased high-order correlations was mentioned in some works on cryo-electron microscopy (Lan *et al.*, 2022 ▸; Bendory, Khoo *et al.*, 2023 ▸). However, unlike XFEL imaging, the noise in cryo-electron microscopy occurs in the measurement of 2D real-space projections, whereas correlations are computed to characterize the Fourier space. Consequently, the deduction of the impact of noise on correlations would require more complex approaches.

After eliminating the shot noise and the detector noise, the obtained correlations *C*_s_, *T*_s_ and *D*_s_ may be used for reconstructing a 3D diffraction intensity volume. In this approach, the contaminating 3D background caused by *I*_Compt_(*k*) and *I*_inst_(*k*) need to be subtracted before performing phase retrieval. Alternatively, the background in correlations can be subtracted directly from the correlations prior to 3D reconstruction. As discussed in Appendices *A*1[App appa] and *A*2[App appa], *I*_Compt_(*k*) and *I*_inst_(*k*) are assumed to be isotropic and can be either estimated or measured separately. We use *I*_b_(*k*) to denote the total background as *I*_b_(*k*) = *I*_Compt_(*k*) + *I*_inst_(*k*). After background subtraction, the target correlations *C*, *T* and *D* for the coherent scattering intensity from the sample, *I*(**k**), can be obtained. The equations for background subtraction are given as follows:

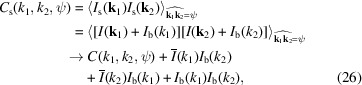




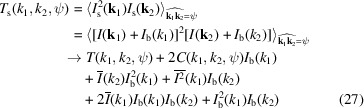

and

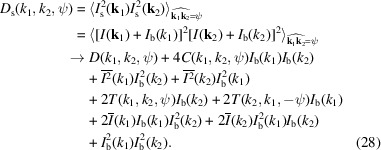

In equations (26)–(28) [Disp-formula fd28], 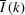 is the angular average of the coherent scattering intensity *I*(**k**). It can be obtained by correcting 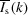 in equation (24):

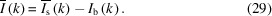




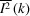
 is the angular average of the square of the coherent scattering intensity *I*(**k**). It can be obtained by correcting 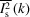 in equation (25):

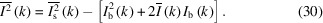

Here, we assume that the instrument background is isotropic. However, in certain scenarios, such as parasitic scattering at upstream components, the instrument background can become anisotropic. In such situations, it becomes necessary to make corresponding adjustments to equations (26)–(28) [Disp-formula fd28].

At the end, after noise elimination and background subtraction, the obtained correlations *C*, *T* and *D* for the coherent scattering signals *I*(**k**) are put into the same reconstruction pipeline as mentioned in Sections 2.1 and 2.2.

## 3. Results

### 3.1. Structure determination and resolution

As a demonstration, we reconstruct the structure of yeast nucleolar pre-60S ribosomal subunit (PDB ID 6c0f; Sanghai *et al.*, 2018 ▸). Its electron-density model in real space is calculated with *Chimera* (Pettersen *et al.*, 2004 ▸), having 199^3^ voxels and a voxel size of 0.4 nm. We simulate 10 000 diffraction patterns at random orientations.

The Ewald sphere is assumed to be flat. Photon shot noise is not introduced as a first demonstration. Following equations (2), (12) and (8), we compute the double, triple and quadruple correlations *C*(*k*_1_, *k*_2_, ψ), *T*(*k*_1_, *k*_2_, ψ), and *D*(*k*_1_, *k*_2_, ψ) from the 10 000 diffraction patterns.

**Figure 1:**
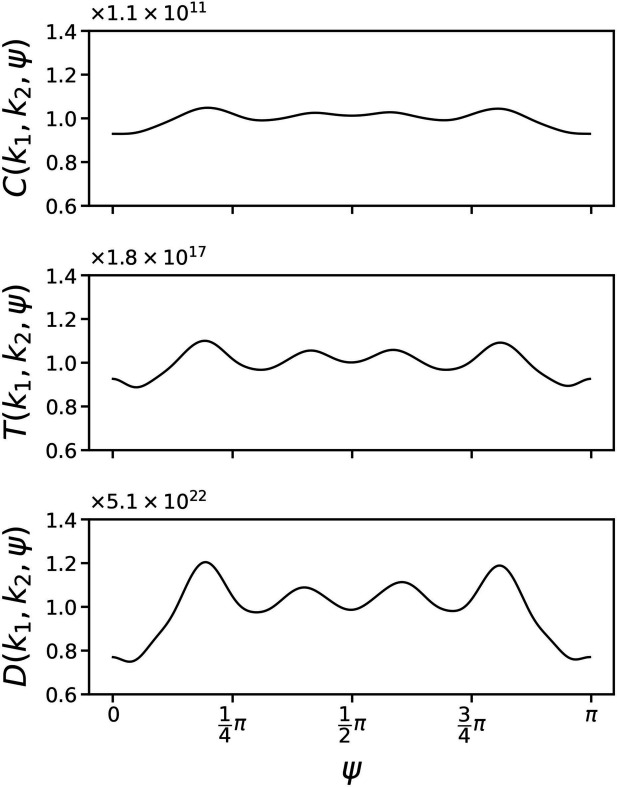
Double correlations *C*(*k*_1_, *k*_2_, ψ), triple correlations *T*(*k*_1_, *k*_2_, ψ) and quadruple correlations *D*(*k*_1_, *k*_2_, ψ) at (*k*_1_, *k*_2_) = (0.15 nm^−1^, 0.25 nm^−1^), as a function of ψ. They are computed from 10 000 simulated diffraction patterns.

As Friedel’s law ensures that the 3D diffraction intensity volume is symmetric, we only need to compute the correlations in the range ψ ∈ [0, π). In addition, when the number of diffraction patterns of random orientations is large enough, the correlations should converge to be symmetric about ψ = π/2 according to equation (2). To illustrate, Fig. 1 ▸ plots three orders of correlations at *k*_1_ = 0.15 nm^−1^ and *k*_2_ = 0.25 nm^−1^. The correlations of different orders display similar oscillatory patterns, but with different amplitudes. In practical experiments, these oscillations may indicate the basic symmetry of the sample (Wochner *et al.*, 2009 ▸) and serve as a promising clue to check the validity of experimental data prior to reconstruction.

Following equation (4), we obtain partial correlation matrices **C**_*l*_ with setting *l*_max_ to 30. Next, we obtain basis vectors *c*_*l*,*i*_ *u*_*l*,*i*_(*k*) through eigen decomposition of **C**_*l*_. As the correlations should be symmetric about ψ = π/2, all elements in **C**_*l*_ should be zero when *l* is odd. This is because 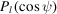 in equation (4) is an odd function with respect to ψ = π/2 when *l* is odd, and an even function with respect to ψ = π/2 when *l* is even. Therefore, there are no basis vectors in odd *l* subspaces.

**Figure 2:**
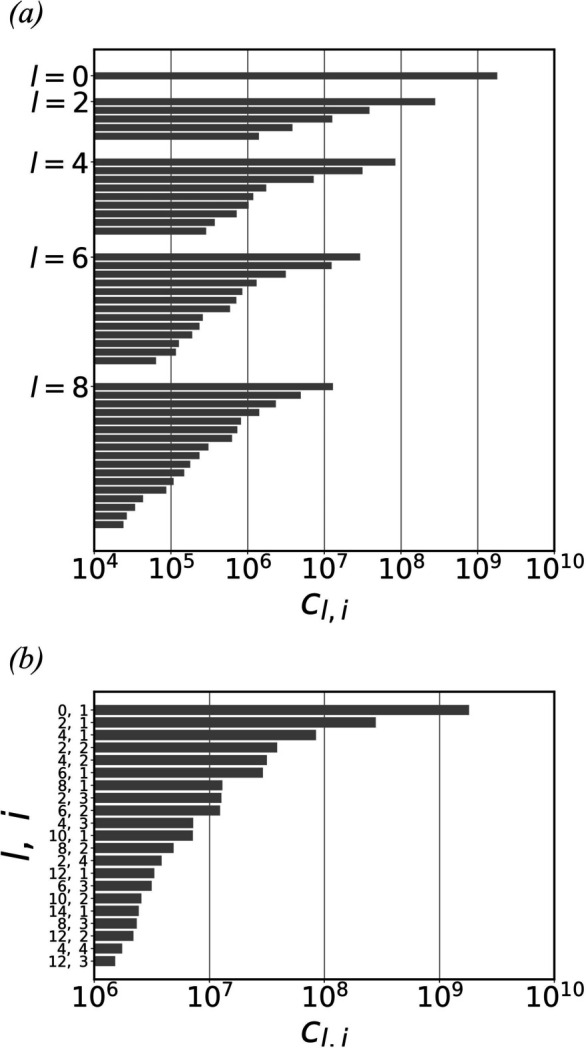
(*a*) Norms of all basis vectors in the subspaces of *l* no larger than 8. There are no basis vectors in odd *l* subspaces. (*b*) The top 21 basis vectors with the greatest norms among all basis vectors in the subspaces of *l* no larger than 30. The term *l* denotes the degree of the subspace and *i* represents the *i*th basis vector in that subspace.

Fig. 2 ▸(*a*) visually represents the norms *c*_*l*,*i*_ of all basis vectors in the subspaces of *l*, no larger than 8. It is clear that their magnitudes have a significant variation. This motivates the idea of employing only those basis vectors with large norms and ignoring those with small norms. For this reason, we sort all 495 basis vectors in the subspaces of *l* no larger than 30 according to their norms, from the maximum to the minimum. The norms *c*_*l*,*i*_ of the top 21 basis vectors are shown in Fig. 2 ▸(*b*), with the corresponding degree *l* and order *i* marked on the left side. Among these 21 basis vectors, the maximum degree *l* is 14. Based on the method of constructing unitary matrix 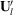 in Section 2.2, we need to optimize 1176 independent variables to align all basis vectors in the subspaces of *l* no larger than 14. However, as we only align the top 21 basis vectors, this number is reduced to 250.

To demonstrate how the model’s basic shape is dominated by a small number of basis vectors with larger norms, we extract the 3D diffraction intensity volume corresponding to the basis vectors of interest and recover the corresponding real-space density model by phase retrieval. To do this, we start by calculating the set of unitary matrices **U**_*l*_ using the known SH coefficients *I*_*l*,*m*_(*k*) of the original model, based on an equation similar to equation (10). Next, we extract the 3D diffraction intensity volume using equation (6) with setting all other basis vectors to zero. Details of the phase retrieval are presented in Appendix *A* [App appa].

**Figure 3:**
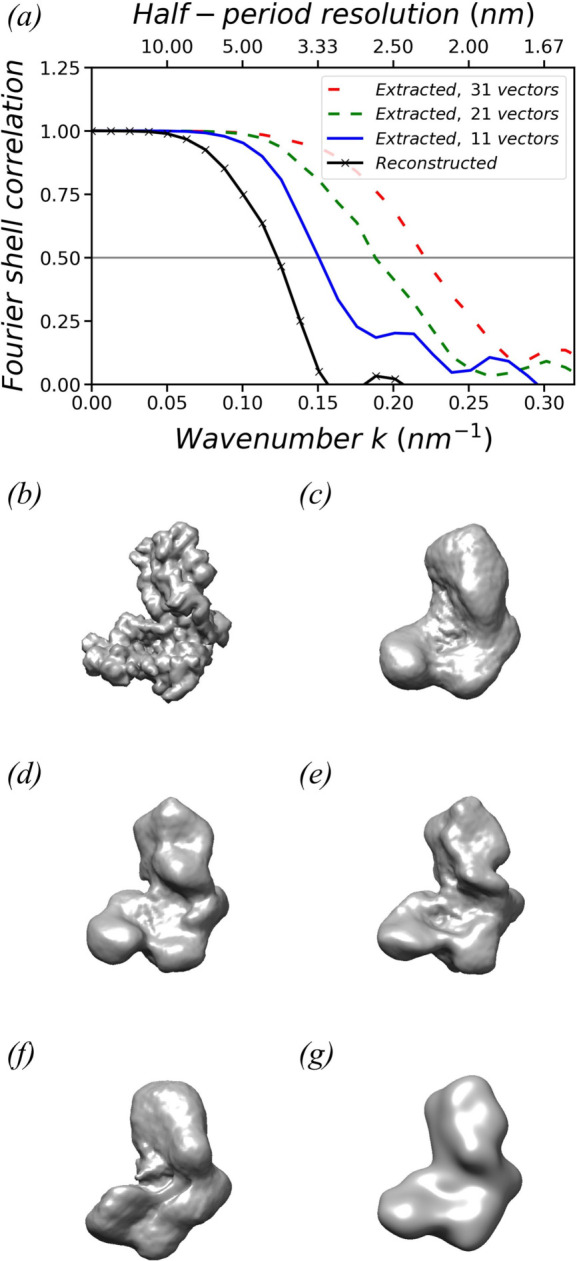
(*a*) FSCs of the extracted models and the reconstructed model with respect to the original model. (*b*) The original model of a yeast nucleolar pre-60S ribosomal subunit. (*c*), (*d*) and (*e*) show the extracted models corresponding to the top 11, 21 and 31 basis vectors with the largest norms, respectively. Their half-period resolutions are 3.32, 2.66 and 2.26 nm, respectively. (*f*) The model reconstructed from 10 000 simulated diffraction patterns by aligning the top 21 basis vectors. Its half-period resolution is 4.07 nm. (*g*) A low-resolution model obtained by applying a 4 nm FWHM Gaussian filter to the original model. In (*b*)–(*g*), the isosurfaces are plotted at 10% of the maximum density.

Figs. 3 ▸(*c*), 3 ▸(*d*) and 3 ▸(*e*) show the extracted models corresponding to the top 11, 21 and 31 basis vectors with the largest norms, respectively. To align the basis vectors, 89, 250 and 381 independent variables need to be optimized, respectively. Fig. 3 ▸(*a*) plots the Fourier shell correlations (FSCs) between the extracted models and the original model. The corresponding half-period resolutions were determined to be 3.32 nm, 2.66 nm and 2.26 nm, respectively, by reading out the critical *k* value at the threshold of 0.5. As expected, the inclusion of additional basis vectors provides more detail.

Next, the reconstruction using our progressive iterative optimization algorithm is performed. We employ the top 21 largest basis vectors as alignment subjects. To mitigate the impact of optimization convergence toward local minimum, we run 20 independent trials, each starting with varying random initial values of the parameters, and then average the results of the optimized 3D diffraction intensity volumes. The final optimization errors are discussed in Section S1. The runtime for a single optimization process typically takes 1 h on a consumer-grade laptop equipped with an Intel Core i5-7300U processor (2.6 GHz, 2 cores). To run 20 independent optimizations in parallel, we use a workstation with dual Intel Xeon Gold 6336Y processors (2.4 GHz, 48 cores in total), and the runtime remains ∼1 h.

The reconstructed density model is obtained after phase retrieval, as shown in Fig. 3 ▸(*f*). Its FSC with respect to the original model is plotted in Fig. 3 ▸(*a*), indicating a half-period resolution of 4.07 nm, which is ∼12.7% of the model’s 32 nm diameter. The resolution difference between the reconstructed model and the extracted model, both using the top 21 greatest basis vectors, uncovers the limit of the current progressive iterative optimization algorithm. Nevertheless, despite the limited resolution, the reconstructed model reveals the basic shape of the original model. This impression can be reinforced by comparing the reconstructed model with a low-resolution model obtained by applying a 4 nm full width at half-maximum (FWHM) Gaussian filter to the original model, as shown in Fig. 3 ▸(*g*). The method to average the optimized 3D diffraction intensity volumes is described in Appendix *B* [App appb]. The method of phase retrieval is described in Appendix *C* [App appc].

### 3.2. Performance under simulated experimental conditions

To evaluate our method’s performance under practical experimental conditions, we simulate a total of 50 000 diffraction patterns, with the inclusion of Compton scattering background, instrument background, photon shot noise and detector noise. The instrument background in a pattern is set to 5% of the coherent scattering intensity. Three different incident laser fluxes of *I*_0_ = 10^12^, 10^13^ and 10^14^ photons per square micrometre per shot are considered. The detector is assumed to be pnCCD (Strüder, 2000 ▸). Detailed explanations on the background and noise are presented in Appendix *A* [App appa]. In this simulation, the incident wavelength is set to 1 nm, equivalent to an X-ray photon energy of 1239.8 eV. The density model in real space consists of 80^3^ voxels with a voxel size of 2.5 nm. The wavenumber at the edge of the diffraction patterns is 0.2 nm^−1^. We still assume a flat Ewald sphere. Examples of the simulated patterns are displayed in Fig. S2 in Section S2 of the supporting information.

**Figure 4:**
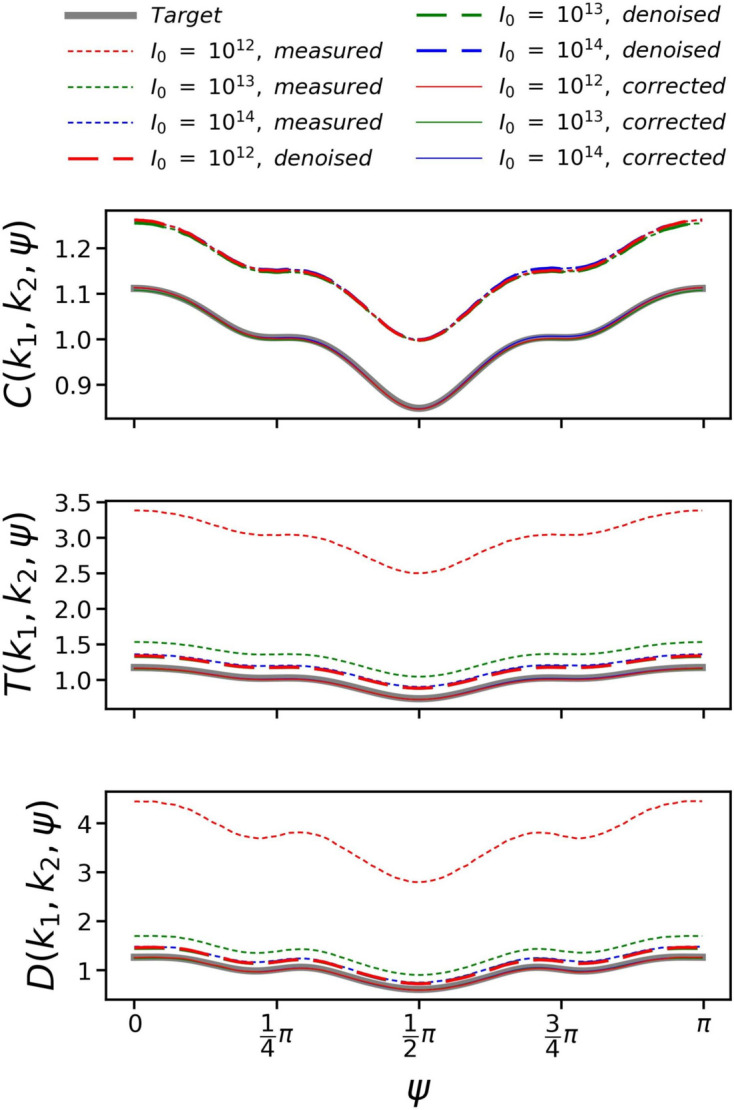
Double, triple and quadruple correlations at (*k*_1_, *k*_2_) = (0.12 nm^−1^, 0.08 nm^−1^). The thick gray lines represent the target correlations calculated directly from the original model. The short-dashed red, green and blue lines represent the correlations *C*_d_, *T*_d_ and *D*_d_ computed from 50 000 simulated diffraction patterns including background and noise, with the incident laser flux set to 10^12^, 10^13^ and 10^14^ photons per square micrometre per shot, respectively. The long-dashed lines are the corresponding correlations *C*_s_, *T*_s_ and *D*_s_ after noise elimination. The thin solid lines are *C*, *T* and *D* after further background subtraction, which are less noticeable as they are overlapped with the thick gray lines.

Fig. 4 ▸ plots the correlations *C*_d_, *T*_d_ and *D*_d_ computed from all simulated diffraction patterns, at *k*_1_ = 0.12 nm^−1^ and *k*_2_ = 0.08 nm^−1^. For ease of comparison, the target correlations are calculated directly from the original model and plotted. Additionally, the correlations *C*_s_, *T*_s_ and *D*_s_ after noise elimination, as well as the correlations *C*, *T* and *D* after further background subtraction, are also plotted. It is evident that the final corrected correlations *C*, *T* and *D* overlap well with their respective target correlations, demonstrating the effectiveness of our method for noise elimination and background subtraction. The gaps between *C*_s_, *T*_s_ and *D*_s_ and their respective target correlations represent the impact of background. Similarly, the gaps between *C*_d_ and *C*_s_, *T*_d_ and *T*_s_, and *D*_d_ and *D*_s_ represent the impact of noise. According to equation (21), for double correlations, since the mean of the noise is zero, uncorrelated noise has been canceled out. The impact of noise is zero, and therefore noise elimination is not needed. This is represented by the complete overlap of *C*_d_ and *C*_s_ in Fig. 4 ▸. In contrast, for triple and quadruple correlations, we can visually observe the impact of noise, and therefore noise elimination is essential. The impact of noise is dominated by the incident laser flux *I*_0_. When *I*_0_ = 10^14^, *T*_d_ and *D*_d_ exhibit only minor deviations from *T*_s_ and *D*_s_. However, as *I*_0_ decreases to 10^13^ and 10^12^, the deviations increase, indicating a greater susceptibility to noise.

**Figure 5:**
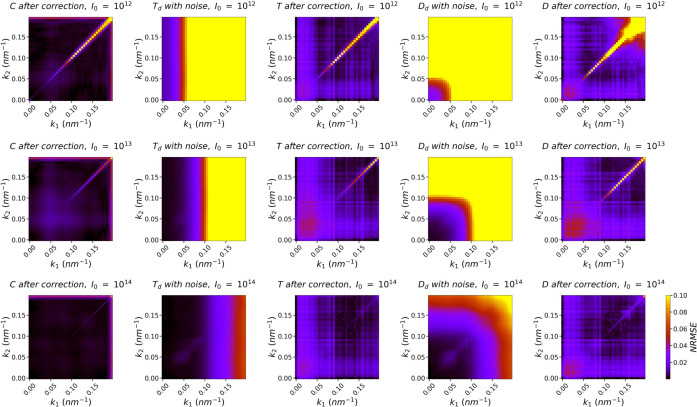
NRMSE between the final corrected correlations *C*, *T* and *D* and their respective target correlations at all (*k*_1_, *k*_2_) pairs. The NRMSE between *T*_d_ and *T*_s_, as well as *D*_d_ and *D*_s_, is also plotted to visualize the impact of noise.

To quantify the deviations between two correlations at the same (*k*_1_, *k*_2_) pair, we calculate the root mean square error (RMSE) following equation (S1) in Section S3 of the supporting information. Since the diffraction intensities in low *k* and high *k* regions have orders of magnitude difference, it is necessary to normalize the RMSE values to describe their relative impact on the correlations in different *k* regions. The normalization factor at (*k*_1_, *k*_2_) is determined to be the magnitude of the target correlation, which is calculated following equation (S2). Finally, the normalized RMSE (NRMSE) values at all (*k*_1_, *k*_2_) pairs, calculated via equation (S3), are visually depicted in Fig. 5 ▸. The second and fourth columns in the figure display the normalized deviations between *T*_d_ and *T*_s_, and between *D*_d_ and *D*_s_, respectively, illustrating the impact of noise. The impact of noise appears acceptable at *I*_0_ = 10^14^, but becomes apparently large from *k* = 0.10 nm^−1^ when *I*_0_ = 10^13^ and from *k* = 0.05 nm^−1^ when *I*_0_ = 10^12^. This suggests that the reliable range of *k* for high-order correlations is severely limited by a weak incident laser flux without noise elimination. In other words, our noise elimination is the key to enabling the present reconstruction method using high-order correlations to be potentially feasible with practical experimental data.

As a comparison, we also plot the normalized deviations between the final corrected correlations (*C*, *T*, *D*) and their respective target correlations. Clearly, after noise elimination and background subtraction, the deviations become much smaller. This is consistent with the overlap of correlations at (0.12 nm^−1^, 0.08 nm^−1^) in Fig. 4 ▸.

Even after noise elimination and background subtraction, a noticeable deviation is still present at *k*_1_ = *k*_2_ for all *C*, *T* and *D*. This deviation locates at ψ = 0, *i.e.* **k**_1_ = **k**_2_, where the noise, Δ_s_(**k**_1_) + Δ_d_(**k**_1_) and Δ_s_(**k**_2_) + Δ_d_(**k**_2_), is self-correlated and cannot be canceled out, as derived in equations (21)–(23) [Disp-formula fd23]. To mitigate this effect, we exclude the data points around ψ = 0 from our analysis. This reduces the number of ψ angle points, namely the number of sub-equations in equation (4). However, since the number of sub-equations is still much greater than the number of unknowns, this exclusion does not affect the subsequent analysis.

**Figure 6:**
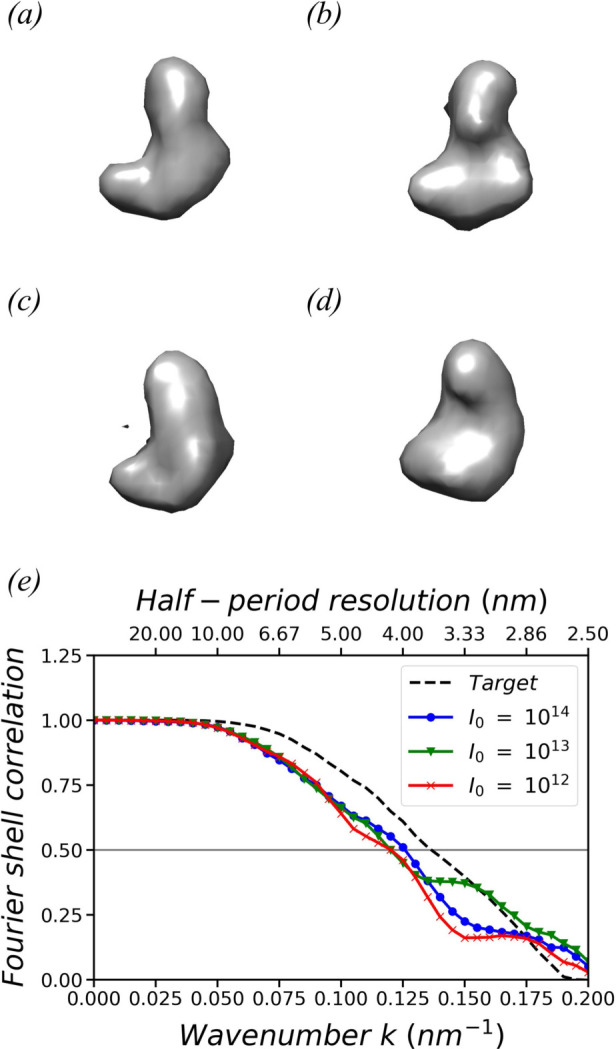
(*a*) A model structure reconstructed using the target correlations. (*b*), (*c*) and (*d*) Model structures reconstructed from 50 000 simulated diffraction patterns at *I*_0_ = 10^14^, 10^13^ and 10^12^ photons per square micrometre per shot, respectively. The simulated diffraction patterns contain background and noise. (*e*) shows the FSCs between the reconstructed models and the original model. In (*a*)–(*d*), the isosurfaces are plotted at 10% of the maximum density.

Finally, we put the corrected correlations (*C*, *T* and *D*) into the same reconstruction pipeline as mentioned in Section 3.1. To distinguish errors arising from the progressive iterative optimization process, we also make a reconstruction using the target correlations, as shown in Fig. 6 ▸(*a*). The models reconstructed from the simulated diffraction patterns at *I*_0_ = 10^14^, 10^13^ and 10^12^ are presented in Figs. 6 ▸(*b*), 6 ▸(*c*) and 6 ▸(*d*), respectively. We plot their FSCs with respect to the original model. Their half-period resolutions are 3.66, 3.97, 4.17 and 4.16 nm, respectively. Despite the slightly lower resolutions, all three reconstructions from the simulated diffraction patterns reveal the basic shape of the original model, demonstrating the effectiveness of our method for noise elimination and background subtraction, as well as the robustness of our reconstruction method under simulated experimental conditions. In Fig. 6 ▸, the reconstructed models appear smeared simply due to their large voxel size of 2.5 nm.

To assess the requirements of the number of diffraction patterns for the convergence of high-order correlations in the presence of noise, we evaluate the performance of our reconstruction with different numbers of patterns – specifically, 50 000, 5000 and 500 simulated patterns. The incident laser flux *I*_0_ is set to 10^13^ and 10^12^ photons per square micrometre per shot. For simplicity, here we add only photon shot noise, which is the major source of noise, to the simulated patterns. Fig. S5 in Section S4 plots the target correlations and the corrected correlations computed from the simulated patterns at *k*_1_ = 0.12 nm^−1^ and *k*_2_ = 0.08 nm^−1^. Overall, the corrected correlations agree with their respective target correlations. At *I*_0_ = 10^13^, when the number of patterns is 500 or 5000, the corrected correlations have small fluctuation errors, and the fluctuation errors become negligible when the number is increased to 50 000. This observation is consistent with the NRMSE colormap at all (*k*_1_, *k*_2_) pairs in Fig. S6. Fig. S7 presents the reconstructed models and their FSCs with respect to the original model. The half-period resolutions using 50 000, 5000 and 500 patterns are 4.07, 4.26 and 4.14 nm, respectively. This small resolution difference suggests that, in our method, although the number of patterns is the larger the better, when the incident laser flux has sufficient intensity, a smaller number, such as 500, can still be acceptable for the convergence of high-order correlations and reasonable reconstruction. At *I*_0_ = 10^12^, employing 50 000 patterns is sufficient to achieve convergence in high-order correlations and produce a successful reconstruction with a resolution of 3.99 nm. In contrast, using 5000 patterns results in a roughly acceptable reconstruction but it has a poorer resolution of 4.63 nm. This implies that, when the incident laser flux has a weaker intensity of *I*_0_ = 10^12^, the required number of patterns falls within the range of 5000–50 000.

### 3.3. Reconstruction from partial diffraction patterns

**Figure 7:**
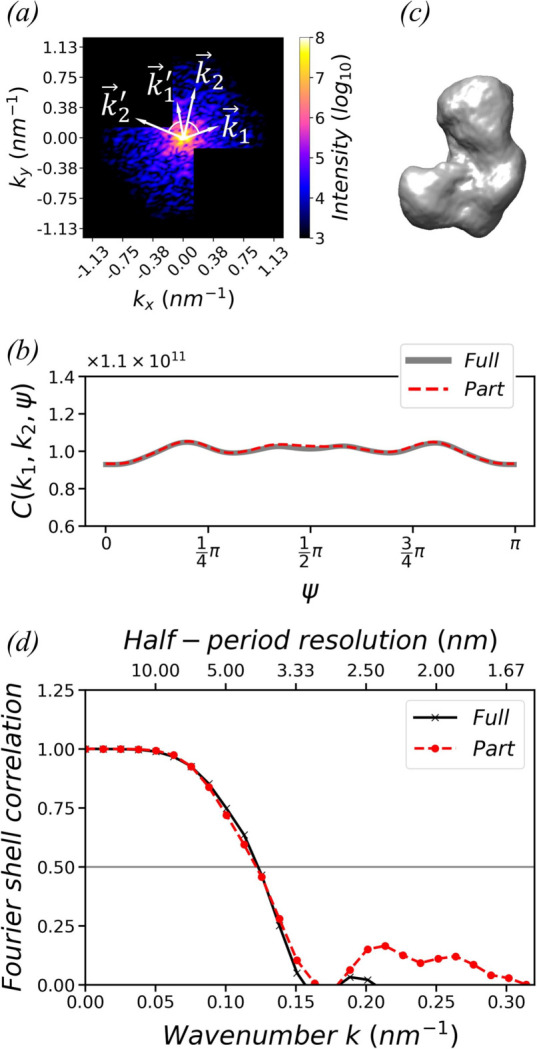
(*a*) A simulated diffraction pattern with two large corners in the second and fourth quadrants removed. (*b*) Double correlations at (*k*_1_, *k*_2_) = (0.15 nm^−1^, 0.25 nm^−1^) computed from 10 000 full diffraction patterns and 10 000 partial diffraction patterns. (*c*) The model reconstructed from partial patterns. The isosurface is plotted at 10% of the maximum density. (*d*) FSCs of the model in (*c*), reconstructed from partial patterns, and the model in Fig. 3 ▸(*f*), reconstructed from full patterns, with respect to the original model.

When computing correlations *C*(*k*_1_, *k*_2_, ψ) from a diffraction pattern via equation (2), one takes an average over all available (**k**_1_, **k**_2_) pairs, with the included angle from **k**_1_ to **k**_2_ being ψ. This does not require **k**_1_ or **k**_2_ to form a complete circle in the diffraction pattern. In other words, the recorded diffraction pattern can be incomplete, with some areas missing. For example, Fig. 7 ▸(*a*) shows a simulated diffraction pattern with two large corners in the second and fourth quadrants removed. When both **k**_1_ and **k**_2_ are located within the remaining area, the (**k**_1_, **k**_2_) pair is included in the computation of correlations. However, if either 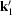 or 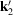 are located within the missing area, the (**k**_1_, **k**_2_) pair is excluded from the computation. In this way, the correlations *C* can still be computed from a set of partial diffraction patterns.

To illustrate, we remove the same two corners in all 10 000 simulated diffraction patterns that are used to reconstruct the model in Fig. 3 ▸(*f*). Fig. 7 ▸(*b*) shows the double correlations *C*(*k*_1_, *k*_2_, ψ) at *k*_1_ = 0.15 nm^−1^ and *k*_2_ = 0.25 nm^−1^ computed from the partial diffraction patterns. They overlap well with the correlations *C* in Fig. 1 ▸, which are computed from the full diffraction patterns.

Next, we obtain partial correlation matrices **C**_*l*_ for 0 ≤ *l* ≤ *l*_max_ by solving the overdetermined linear system in equation (4). Even at the same (*k*_1_, *k*_2_) position, for different ψ values, the number of available 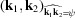 pairs being averaged is different. This suggests that the reliability of different sub-equations is different. To account for this, we denote the total number of available 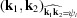 pairs as *N*_*i*_ and apply a weighting factor of (*N*_*i*_)^1/2^ (Aster *et al.*, 2013 ▸) to the *i*th sub-equation of *C*(*k*_1_, *k*_2_, ψ_*i*_). After computing *T* and **T**_*l*_ and *D* and **D**_*l*_ in a similar way, we employ the same reconstruction pipeline as mentioned in Section 3.1.

Fig. 7 ▸(*c*) shows the model reconstructed from the partial diffraction patterns. Its FSC with respect to the original model is plotted in Fig. 7 ▸(*d*). When compared with the FSC of the model reconstructed from full diffraction patterns in Fig. 3 ▸(*a*), it is evident that their resolutions are quite similar. This demonstrates the feasibility of using the correlation-based approach to process incomplete partial diffraction patterns.

## 4. Discussion

In the correlation-based approach, the 3D diffraction intensity volume is constructed based on basis vectors obtained from double correlations from the diffraction patterns. However, since each basis vector can be freely aligned, additional constraints are required to narrow down the general solution to particular solutions of the volume. In our method, we characterize not only the 3D diffraction intensity volume using double correlations but also the 3D squared diffraction intensity volume using quadruple correlations. The internal constraint between the double and quadruple correlations allows us to find the particular solutions. In order to define an objective function that is numerically optimizable, we introduce triple correlations as the bridge between the other two correlations. As a comparison, in Donatelli’s MTIP method (Donatelli *et al.*, 2015 ▸), the additional constraint is a prior knowledge of the sample in real space, such as compact support, symmetry, or upper or lower density bounds. Therefore, it requires simultaneous optimization of the 3D diffraction intensity volume and the corresponding phases. One advantage of this method is that it prevents errors committed during 3D reconstruction from being locked or magnified in phase retrieval (Donatelli *et al.*, 2017 ▸). However, it underutilizes the full potential of the set of diffraction patterns itself to reconstruct the 3D diffraction intensity volume independently. In Ardenne’s method (von Ardenne *et al.*, 2018 ▸), the additional constraint comes from three-photon correlations, which involve three different points in a diffraction pattern. However, as the inversion from three-photon correlations to 3D diffraction intensity volume cannot be analytically expressed, one has to estimate the likelihood of observing the experimental three-photon correlations from a tentative 3D diffraction intensity volume in every iteration, which is computationally expensive.

In the MTIP method and Ardenne’s method, all basis vectors in the subspaces of *l* below a certain *l*_max_ are employed. This is mathematically natural, considering the approach undertaken to iteratively project or randomly rotate the input unitary matrices 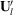 . In our method, we only employ a limited number of basis vectors with large norms. This significantly reduces the number of independent variables to be optimized, while it is still sufficient to preserve the basic shape of the sample. As a consequence, we can convert the problem of 3D reconstruction into a process of minimizing an error function, which has an acceptable number of independent variables. This allows us to take advantage of well established minimization algorithms, such as the Levenberg–Marquardt algorithm or the trust region reflective algorithm (Branch *et al.*, 1999 ▸), as well as existing packages. With the use of a progressive optimization strategy, we expect to achieve a satisfactory convergence robustly. On the other hand, since the aforementioned algorithms are local optimization algorithms, the sought solution may have small perturbations around the theoretical particular solutions. As the number of independent variables to be optimized increases, the impact of getting trapped at a local minimum becomes severer. This has restricted us from employing a larger number of basis vectors in the reconstruction, which is the primary reason limiting the achievable resolution. In the future, it would be beneficial to implement some global optimization algorithms, such as the Monte Carlo simulated annealing, which is also utilized in Ardenne’s method. However, achieving the desired performance with this algorithm requires careful design and adjustment of parameters, including the energy function, temperature and step size. Therefore, we excluded uncertainties arising from parameter adjustments during the initial stage of establishing our reconstruction method.

Currently, the limited SNR in individual diffraction patterns presents a major challenge for XFEL single-particle imaging and the correlation-based approach offers a natural solution. By computing and averaging correlations over all diffraction patterns, this approach effectively accumulates signals and improves the overall SNR, making it particularly suitable for processing experimental data with a poor SNR in each pattern. In all correlation-based approaches when computing the double correlations, and in Ardenne’s method when computing the three-photon correlations, the uncorrelated noise at different points is automatically averaged out and therefore no further correction is needed. This is also a reason for the effectiveness of Ardenne’s method in processing extremely sparse diffraction patterns. In our method, when computing the triple and quadruple correlations, since we square the measured intensity at the same point, the impact of noise remains. We investigated the impact of various sources of noise on high-order correlations. When high-order correlations are converged by collecting sufficient diffraction patterns, the impact of noise can be predicted and subsequently eliminated. To achieve this, we only need to know the second moment of the noise, regardless of whether its probability distribution is Poisson, Gaussian or even pseudo-Voigt. This idea of noise elimination is the key to making our reconstruction method potentially applicable to practical experimental data. Even if the experimental data contain fluctuations and disturbances from other sources, we can still eliminate their effects using an approach similar to equations (22) and (23), as long as we can model them and estimate their second moment. On the other hand, as it has been pointed out, in the presence of noise, high-order correlations are difficult to measure (Kirian, 2012 ▸) or need many patterns to converge (Singer, 2019 ▸). In our simulation, the fluctuation errors of high-order correlations become acceptable with only 500 patterns when the incident laser flux is 10^13^ photons per square micrometre per shot. Even with a laser flux of 10^12^, which is readily achievable in many XFEL facilities, 50 000 patterns are sufficient. Furthermore, with the advent of megahertz single-particle imaging in recent years (Sobolev *et al.*, 2020 ▸), it may no longer be difficult to collect a large number of diffraction patterns to obtain perfect convergence on high-order correlations.

In this work, we assume that we can model the experimental noise with sufficient accuracy. However, in actual experiments, this may not be the case, as the noise may have unexpected sources and its behavior may be too complex to predict. For example, the detector response may change with time and working conditions. In cases where the detector comprises multiple modules, there may be high correlations among the detector noise in these modules. Additionally, charge sharing and noise correlation between adjacent pixels also have an impact. When the diffraction signals from the sample are weak, all these factors can significantly increase the complexity of image processing. This remains a substantial challenge for all 3D reconstruction methods and should be carefully investigated case by case when processing actual experimental data. Specifically, for our method using high-order correlations, it may be beneficial to introduce some model-agnostic denoising strategies. For instance, as shown in Fig. 4 ▸, the most significant impact of noise on high-order correlations is the addition of a relatively ψ-invariant constant intensity. Therefore, when expanding correlations into Fourier–Legendre series using equation (4), the noise impact is mainly isolated in the zeroth *l* subspace, which accounts for the radially averaged intensity in the 3D diffraction volume. Since this radially averaged intensity can be roughly estimated using the double correlations that are less influenced by noise, it may be possible to iteratively retrieve the denoised zeroth *l* subspace. Such a model-agnostic denoising strategy may provide an alternative approach to addressing issues related to unknown noise models. Overall, the assumptions about noise and the noise-correction procedures in this work represent just the initial steps in what is necessary. Dealing with actual noisy experimental data depends on specific experimental conditions and still requires further effort.

The correlation-based approach does not require every diffraction pattern to be complete. In some reports, the computation of double correlations is written as 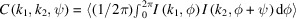 , where *I*(*k*, ϕ) is the diffraction intensity in a pattern and the angle brackets denote an average over all patterns. However, in reality, it is unnecessary for ϕ to cover the full range from 0 to 2π. Even if some parts in each diffraction pattern are missing, we can still measure the spatial correlations and the complete information by collecting many patterns. Such discussions and measurements have been reported before, such as by Clark *et al.* (1983 ▸) and Zaluzhnyy *et al.* (2017 ▸). In the field of XFEL single-particle imaging, the feasibility of processing incomplete partial diffraction patterns is a remarkable feature for the correlation-based approach, whereas for many orientation-based approaches, the missing parts in each pattern should not be large. In practical experiments, missing parts in measured diffraction patterns are often encountered due to various reasons, such as detector gaps between multiple sensor pieces, abnormal pixels or even small dead regions in the detector. On the other hand, accepting incomplete partial diffraction patterns makes it more flexible to arrange instruments in the narrow space between the sample and the detector. In Section 3.3, we demonstrated an extreme case where the majority of the second and fourth quadrants in all diffraction patterns are missing, although practically, the missing parts would be much smaller. Additionally, they are allowed to vary in different diffraction patterns.

Due to the complexity of XFEL image processing, it may be desirable to apply multiple data-analysis approaches to the same experimental dataset, either independently for comparison or sequentially at different processing stages. Our method provides a robust way to produce an *ab initio* 3D intensity reconstruction, which may serve as an unbiased initial model for subsequent iterative refinements to achieve high-resolution structure reconstruction. The combination of a coarse *ab initio* reconstruction followed by an iterative probabilistic reconstruction has been commonly utilized in the field of cryo-electron microscopy (Lan *et al.*, 2022 ▸; Bendory, Khoo *et al.*, 2023 ▸), but is still rare in the processing of XFEL single-particle imaging experimental data. In Section S5, we present preliminary attempts to connect our method with orientation-based approaches by estimating the orientation of every diffraction pattern based on the correlation-reconstructed model. We expect that such a connection can become a beneficial option when analyzing various complex XFEL experimental data.

The correlation-based approach, as noted in its first proposal (Kam, 1977 ▸), can process fluctuation X-ray scattering experimental data, which involves the effects of overlapping diffraction patterns from multiple particles and interparticle interference. It has been proved that the computation of double correlations is not affected by these effects (Pedrini *et al.*, 2013 ▸). However, when computing high-order correlations, corrections are necessary. As the corresponding theory and equations are rather complex, we plan to discuss this separately in future works.

In summary, we have developed a method for reconstructing the 3D density map of a sample from XFEL single-particle diffraction patterns. The sample can have an irregular unsymmetric shape. No prior knowledge of the sample is needed. This *ab initio* 3D reconstruction method works by computing and analyzing different orders of spatial correlations of diffraction intensities. It could relax the requirements for the quality of experimental diffraction patterns, since correlations are calculated over a whole set of diffraction patterns, and thus individual patterns are allowed to have a poor SNR or missing parts. Currently, we are exploring the possibility of extending this method to process experimental data of fluctuation X-ray scattering.

## Supplementary Material

Supporting information. DOI: 10.1107/S2052252523009831/zf5022sup1.pdf

## Appendix A Model of background and noise

### A1. Compton scattering background

The X-ray scattering at the sample includes coherent scattering and Compton scattering. The former is the signal, while the latter is an intrinsic background. Compton scattering cannot be measured separately. However, if the approximate numbers of carbon, nitro­gen and oxygen atoms can be determined by other techniques, we can estimate the intensity and distribution of Compton scattering. This estimation does not rely on prior knowledge of the sample’s 3D structure.

When the X-ray laser is linearly polarized, the differential Compton scattering cross section from an electron is (Matt *et al.*, 1996 ▸)

where *r*_e_ is the classical electron radius, Θ is the scattering angle, ϕ is the azimuth angle with respect to the polarization and *E*′/*E* accounts for the X-ray photon energy loss in Compton scattering:

where *E* is the incident photon energy, *m*_e_ is the electron mass and *c* is the speed of light.

The differential cross section from an atom is

where *S* is the Compton scattering factor (Hubbell *et al.*, 1975 ▸) and *Z* is the atomic number.

With prior knowledge of the numbers of carbon, nitro­gen and oxygen atoms, the differential cross section from a biological sample is estimated as

In equation (A1), the term  is reduced to  if the X-ray laser is unpolarized. If the X-ray laser is linearly polarized but Θ is small enough or the X-ray laser is unpolarized, the distribution of Compton scattering is isotropic, denoted as *I*_Compt_ = *I*_Compt_(*k*).

As we calculated under the simulated experimental conditions in Section 3.2, the intensity of Compton scattering is weaker by seven orders of magnitude than that of coherent scattering in a pattern.

### A2. Instrument background

In XFEL single-particle imaging experiments, the measured patterns may contain scattering backgrounds originating from various sources other than the sample, such as the instruments, the environment or the solution used to preserve the sample. We refer to these backgrounds collectively as instrument background. Depending on the experimental conditions, the intensity and distribution of the instrument background may vary. Here we assume an isotropic distribution of instrument background, which can be separately measured in an experiment without samples.

In the data synthesis for this work, we assume a 2D Gaussian model for the instrument background:

where *I*_total_ represents the total coherent scattering intensity in a pattern and γ controls the overall background-to-signal ratio. We set  and σ_inst_ = 0.04 nm^−1^.

### A3. Photon shot noise

The intensities of coherent scattering *I*, Compton scattering *I*_Compt_ and instrument background *I*_inst_ together form the overall source intensity *I*_s_ to be measured:

In the measurement of *I*_s_, photon shot noise is intrinsically included and the corresponding intensity can be expressed as

where Δ_s_(**k**) is the photon shot noise. The term *I*_Δs_(**k**) follows a Poisson distribution with a mean of *I*_s_(**k**). The expectation of Δ_s_(**k**) is zero:

The expectation of the square of Δ_s_(**k**) is equal to the mean of *I*_s_(**k**):

### A4. Detector noise

The measurement of *I*_Δs_ is also affected by detector noise, including Fano noise, dark current noise, readout noise, photo response non-uniformity, dark signal non-uniformity, *etc*. For an ideal photon-counting detector, the detector noise is zero and can be neglected. Unfortunately, up to now, photon-counting detectors still cannot be used in XFEL imaging experiments due to the inability to handle large instantaneous fluxes. In the case of a conventional photon-integrating detector, the aforementioned detector noise must be taken into account.

In the data synthesis for this work, we assume the use of a pnCCD detector (Strüder, 2000 ▸). Its sensor material is silicon. Its system noise, *n*_e_, which combines dark current noise, readout noise and other electronic noise, is ∼4 e^−^ (RMS). We make the approximation that both the Fano noise and the system noise follow Gaussian distributions. The intensity, *I*_d_, measured by the detector can be expressed as

where Δ_d_ is the detector noise. The term *I*_d_(**k**) follows a Gaussian distribution with a mean of *I*_Δs_(**k**). The standard deviation is

The constants α and β are determined by the detector’s sensor material and electronic noise level, as well as the X-ray photon energy. They are defined as

and

where *F* is the Fano factor and ε is the average energy required to generate an electron–hole pair in the detector sensor. Their values depend on the sensor material. For silicon, we take *F* as 0.115 (Lowe, 1997 ▸) and ε as 3.65 eV (Scholze *et al.*, 1998 ▸). With X-ray photon energy *E* set at 1239.8 eV, we have α = 3.396 × 10^−4^ and β = 1.392 × 10^−4^. Since *F* and ε are invariant physical constants, and *n*_e_ describes the detector system’s electronic noise level, which should be carefully inspected, we assume the precise knowledge of these parameters, along with the derived α and β. Additionally, α and β can also be estimated or verified in experimental diffraction patterns, as discussed in Section S6.

The expectation of Δ_d_ is zero:

The expectation of the square of Δ_d_ is equal to  :

## Appendix B Averaging the optimized 3D diffraction intensity volumes of different orientations

When reconstructing the 3D diffraction intensity volume from correlations, we run 20 independent optimization trials, each starting with varying random initial values of the parameters. The optimized volumes have arbitrary and uncontrolled orientations. Before averaging them, it is essential to carefully align their orientations.

We use **I**_*i*_ to denote the *i*th volume and **I**_aver_ to denote the averaged volume:

where *N*_trial_ is 20 and  is a 3D rotation operator. The deviation between **I**_aver_ and **I**_*i*_ is defined as the square of Euclidean distance:

The rotation angles for all  are selected to minimize the total deviation Δ:

In practice, minimizing the total deviation Δ is difficult when trying to align or find the optimum rotation angles for all the volumes simultaneously. For this reason, the minimization of Δ is approximated using an iterative method. In the first iteration, we set  as **I**_1_ and use it to align each volume by minimizing the corresponding Δ_*i*_ in equation (B2). Then, it is updated to  via equation (B1) for the second iteration. This iterative process is repeated until Δ converges. Typically, we need to iterate five times.

The square of Euclidean distance between two volumes, denoted as **A** and **B**, is defined as

In this work, the 3D diffraction intensity volume is given in the form of SH coefficients. The square of Euclidean distance can be equivalently calculated as

The rotation of the SH coefficients of a 3D volume is given by

where *I*_*l*,*m*_(*k*) and *I*_*l*,*m*′_(*k*) are the SH coefficients of the 3D volumes before and after rotation, respectively, (α, β, γ) are Euler angles of the rotation, and *D*_*l*,*m*′,*m*_ is the element in the corresponding Wigner-**D** matrix. For a 3D volume, rotating its SH coefficients is more efficient than rotating its sampling points in spherical or Cartesian coordinates, as the interpolation of sampling points is not needed.

In equation (B2), when **I**_aver_ and **I**_*i*_ are given, Δ_*i*_ is a non-linear function of the Euler angles (α, β, γ) for rotation. To minimize Δ_*i*_, we employ the trust region reflective algorithm. To find the global minimum, we run abundant trials with randomly chosen initial values of (α, β, γ).

## Appendix C Phase retrieval

We use the hybrid input–output approach (Fienup, 1982 ▸), which iterates a total of 500 times. The β factor is set as 0.8. The initial support is the entire volume, but then it is updated every 20 iterations using the shrink-wrap method (Marchesini *et al.*, 2003 ▸). In the shrink-wrap process, the reconstructed density model is convolved with a Gaussian kernel of width σ, and the isosurface at ε of the maximum density becomes the updated compact support. In this work, we set ε to 5%. The σ value in the first shrink wrap is 3 voxels and is reduced by 0.01 with each subsequent shrink wrapping. In order to reduce the initial value dependency inherent in phase retrieval, we employ a strategy of running 100 independent trials with varying random initial phases and subsequently averaging the reconstructed density models. This strategy allows us to obtain a more robust density reconstruction.

In the 100 independent trials, the reconstructed density models have ambiguities of conjugate flip and translational shift. Alignment is essential before averaging them. Our alignment method is described as follows. To align two models, denoted as ρ_*A*_ and ρ_*B*_, we flip ρ_*B*_ to ρ_*B*′_ and then compute the convolutions of ρ_*A*_*ρ_*B*′_ as well as ρ_*A*_*ρ_*B*_. By comparing the maximum values in these two convolutions, we can determine whether ρ_*A*_ and ρ_*B*_ have a flip relationship or not. The translational shift from ρ_*A*_ to ρ_*B*_ (or ρ_*B*′_) corresponds to the displacement from the coordinate center to the location of the maximum in that convolution. To align the 100 reconstructed density models, we use an iterative method. In the first iteration, we set the first model as the reference to align the other 99 models. In the second iteration, the reference model is updated by averaging the 100 aligned models. Typically, this iteration process is repeated five times.
