# Supplementary material for: Structure determination using high-order spatial correlations in single-particle X-ray scattering
Source: IUCrJ. 2024 Jan 1;11(Pt 1):92–108. doi: 10.1107/S2052252523009831 (PMC10833384; doi:10.1107/S2052252523009831)
Supplement: Supplementary file 1 [file m-11-00092-sup1.pdf]

IUCrJ

Volume 11 (2024)

Supporting information for article:

Structure determination using high-order spatial correlations in single-particle X-ray scattering

Wenyang Zhao, Osamu Miyashita, Miki Nakano and Florence Tama

Structure determination using high-order spatial correlations in single-particle X-ray scattering

Authors

Wenyang Zhao[a], Osamu Miyashita[a]*, Miki Nakano[a] and Florence Tama[abc]*

[a]Computational Structural Biology Research Team, RIKEN Center for Computational Science, 6-7-1 Minatojima-minamimachi, Chuo-ku, Kobe, Hyogo, 650-0047, Japan

[b]Institute of Transformative Bio-Molecules, Nagoya University, Furo-cho, Chikusa-ku, Nagoya, Aichi, 464-8601, Japan

[c]Department of Physics, Graduate School of Science, Nagoya University, Furo-cho, Chikusa-ku, Nagoya, Aichi, 464-8601, Japan

Correspondence email: osamu.miyashita@riken.jp; florence.tama@riken.jp

Supporting Information

S1. Local minimum in optimizing the 3D diffraction intensity volume

To reconstruct the model in Figure 3(f), we conduct 20 independent optimization processes with random initial parameters. The values of the error function $\mathcal{F}$ in Eq. (14) before the optimization are normalized to make their average value equals to unity. For each optimized 3D diffraction intensity volume, we perform phase retrieval, reconstruct the density model, and assess its resolution. The final optimization errors and resolutions are plotted in Figure S1, represented by blue points.

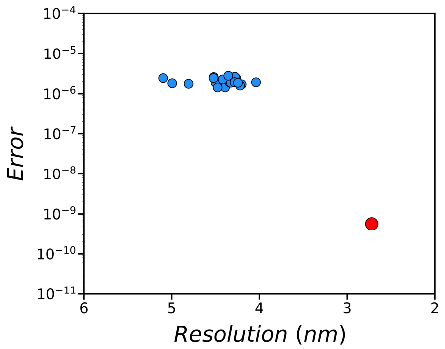

Figure S1 Final optimization errors and resolutions of the corresponding reconstructed models. Blue points: randomly initialized optimizations; red point: the optimization initiated manually near the “answer”.

The reconstructed model in Figure 3(f), obtained by averaging 20 optimized 3D diffraction intensity volumes, has a resolution of 4.07 nm. It surpasses the resolutions achieved in every independent optimization. This illustrates that the approach of averaging is effective in reducing the impact of the optimizer getting trapped at a local minimum.

Currently, the primary factor limiting the achievable resolution is the optimizer's tendency to converge to a local minimum. To verify this, we manually initialize an optimization process from a point near the "answer". The error at this initial point is 5.7×10^{-6}, and the final optimization error is 5.6×10^{-10}, which is three orders of magnitude smaller than the final errors of the randomly initialized optimizations. Correspondingly, the resolution greatly improves to 2.72 nm, approaching the limit of 2.66 nm in Figure 3(d). This demonstrates the validity of the error function $\mathcal{F}$ in Eq. (14) and suggests that the achievable resolution may be further improved in the future by substituting the current local optimization algorithms to a global optimization algorithm.

S2. Diffraction patterns under simulated experimental conditions

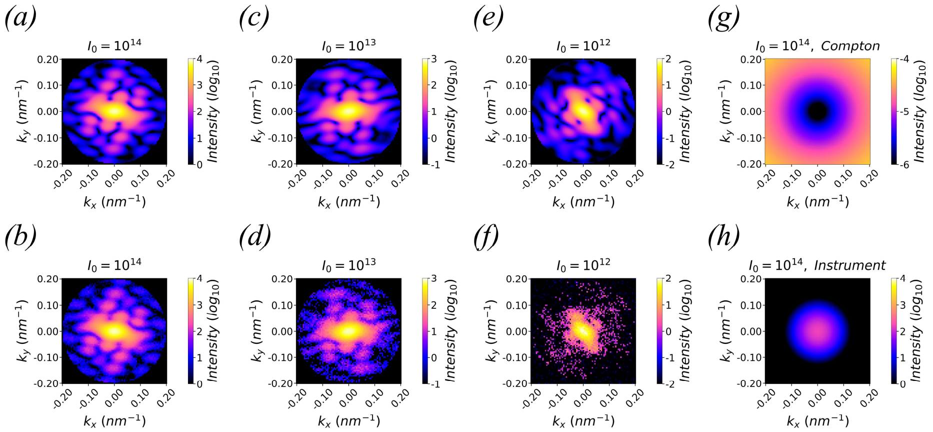

Figure S2 (a), (c), and (e) show examples of the calculated coherent scattering distribution at the incident laser flux I_0 of 10^{14}, 10^{13}, and 10^{12} photons per square micron per shot, respectively. (b), (d), and (f) show the corresponding simulated patterns after adding Compton scattering background, instrument background, photon shot noise, and detector noise. (g) and (h) plot the Compton scattering background and the instrument background at $I_0 = 10^{14}$, respectively. The intensities of the simulated pattern, the Compton scattering background, and the instrument background are approximately 1.2×10^{6}, 1×10^{-1}, and 6×10^{4}, respectively, at $I_0 = 10^{14}$. They decrease proportionally when I_0 decreases to 10^{13} or 10^{12}.

S3. Deviations between simulated experimental correlations and target correlations

The root mean square error (RMSE) between two correlations $C'(k_1, k_2, \psi)$ and $C(k_1, k_2, \psi)$ is defined as follows:

$$RMSE(k_1, k_2) = \sqrt{\frac{1}{\pi}\int_0^{\pi} [C'(k_1, k_2, \psi) - C(k_1, k_2, \psi)]^2 \mathrm{d}\psi}\,. \qquad (S1)$$

The magnitude of the target correlation $C(k_1, k_2, \psi)$ is calculated as:

$$M(k_1, k_2) = \frac{1}{\pi}\int_0^{\pi} C(k_1, k_2, \psi)\mathrm{d}\psi\,. \qquad (S2)$$

Then, the corresponding normalized root mean square error (NRMSE) is defined as:

$$NRMSE(k_1, k_2) = \frac{RMSE(k_1, k_2)}{M(k_1, k_2)}. \qquad (S3)$$

For additional information, similar to Figure 5, the RMSE values for all (k_1, k_2) pairs are visually depicted in Figure S3. The magnitudes of target correlations for all (k_1, k_2) pairs are plotted in Figure S4. Note that the colorbars in Figures S3 and S4 are displayed in logarithmic scale.

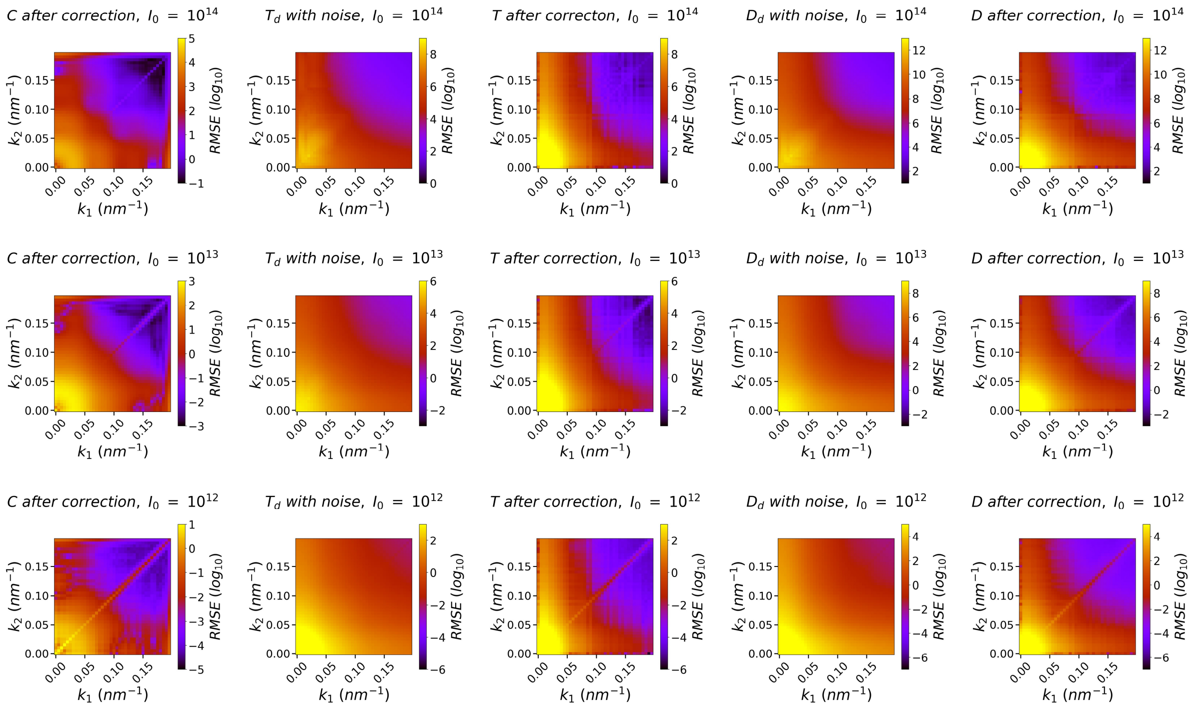

Figure S3 Root mean square error (RMSE) between the final corrected correlations C, T, D, and their respective target correlations at all (k_1, k_2) pairs. The RMSE between T_d and T_s, as well as D_d and D_s, is also plotted to visualize the impact of noise.

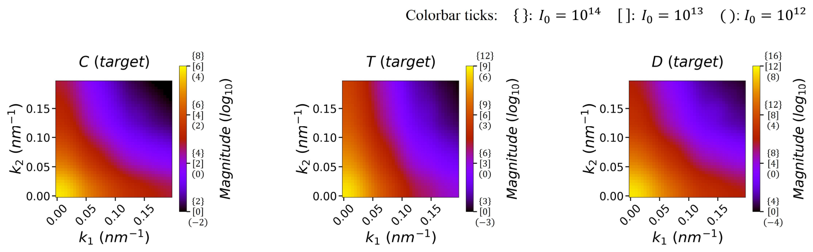

Figure S4 Magnitude of target correlations of C, T, and D at all (k_1, k_2) pairs. The readout of colorbar ticks depends on the I_0 intensity.

S4. Reconstruction with different numbers of diffraction patterns

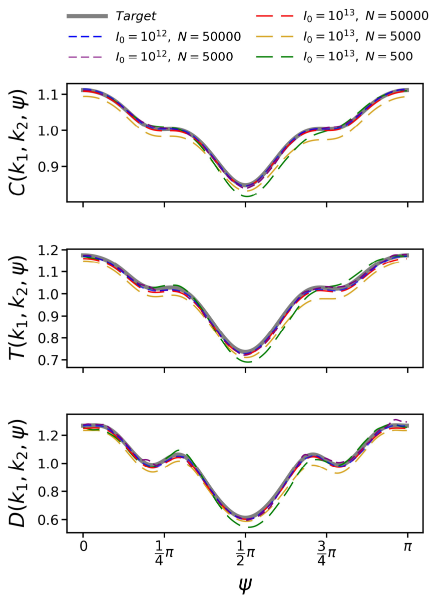

Figure S5 Double, triple, and quadruple correlations at $(k_1, k_2) = (0.12\, nm^{-1}, 0.08\, nm^{-1})$. The thick grey lines represent the target correlations calculated directly from the original model. The dashed lines represent the correlations computed from different numbers of simulated diffraction patterns. The simulated patterns contain photon shot noise, and the incident laser flux I_0 is set to 10^{13} or 10^{12} photons per square micron per shot. The impact of photon shot noise on triple and quadruple correlations has been corrected.

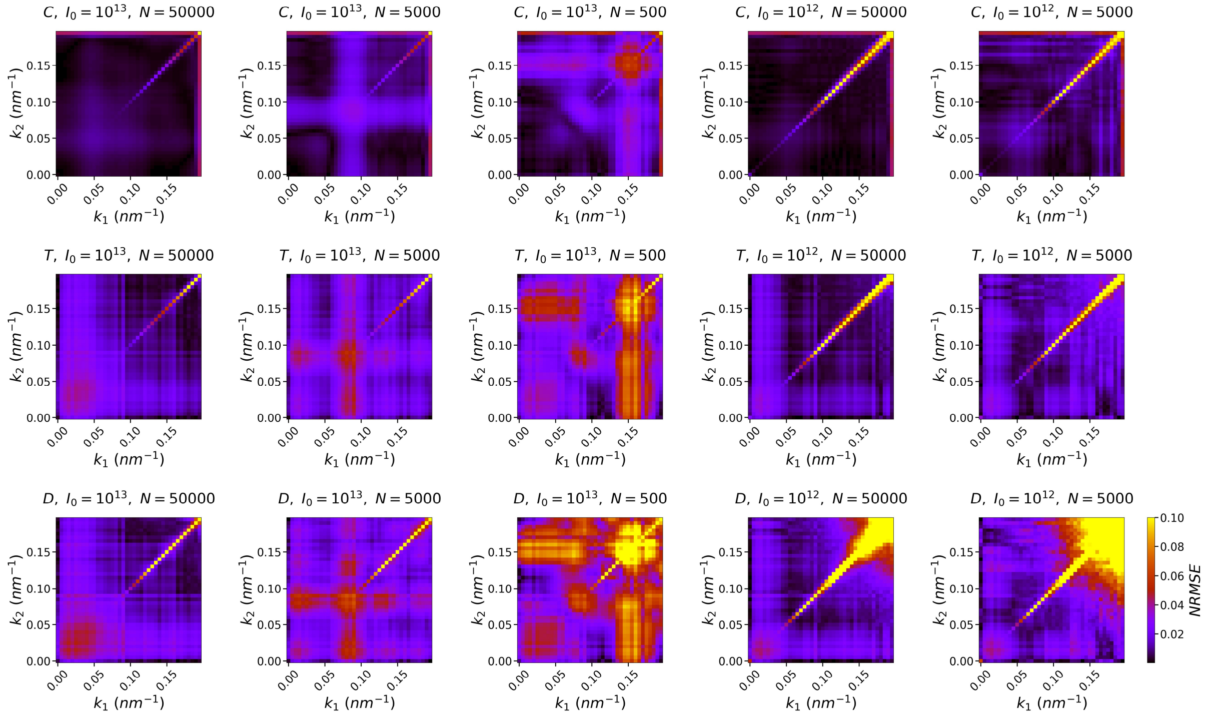

Figure S6 Normalized root mean square error (NRMSE) between the target correlations and the correlations computed from different numbers of simulated diffraction patterns at all (k_1, k_2) pairs. The simulated patterns contain photon shot noise, and the incident laser flux I_0 is set to 10^{13} or 10^{12} photons per square micron per shot. The impact of photon shot noise on triple and quadruple correlations has been corrected.

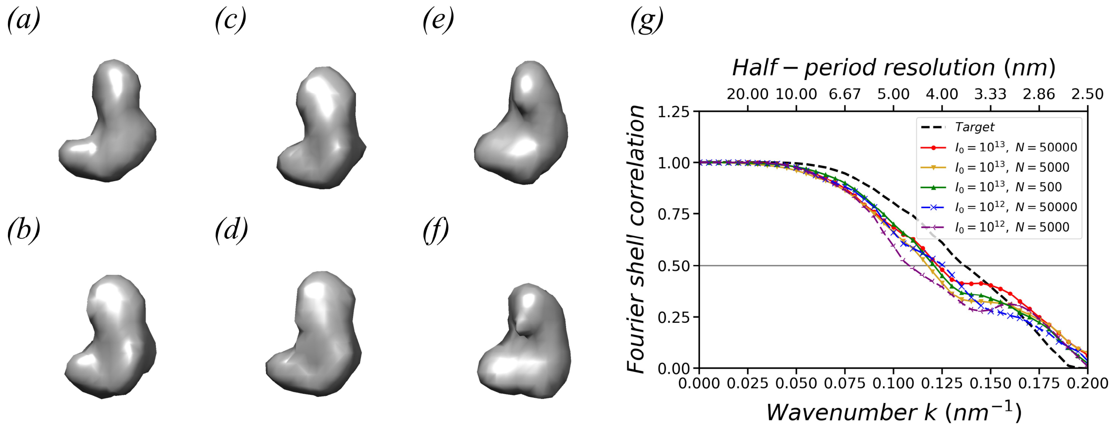

Figure S7 (a) is the model reconstructed using the target correlations. (b), (c), and (d) are the models reconstructed from 50,000, 5,000, and 500 simulated diffraction patterns, respectively, when $I_0 = 10^{13}$. (e) and (f) are the models reconstructed from 50,000 and 5,000 simulated diffraction patterns, respectively, when $I_0 = 10^{12}$. (g) shows the Fourier shell correlations between the reconstructed models and the original model. In (a)-(f), the isosurfaces are plotted at 10% of the maximum density.

S5. Orientation estimation of diffraction patterns using the reconstructed model

In this work, we reconstruct the model by analyzing three orders of correlations computed from all diffraction patterns, without determining the orientation of each pattern. However, after completing the reconstruction, we have the option to incorporate the model to estimate the orientations. The orientation information may prove valuable in other analyses and facilitate comparisons with, as well as connections to, existing orientation-based approaches.

As an illustration, we estimate the orientations of the 10,000 simulated noise-free diffraction patterns, which are used to reconstruct the model in Figure 3(f). We first generate 10,000 reference patterns by slicing the 3D diffraction intensity volume reconstructed by the proposed approach at random orientations. For each simulated diffraction pattern, we find its closest reference pattern and assign its orientation to be that of the reference pattern. Here, the distance between two patterns is defined as the Euclidean distance, namely the root-mean-square error. For instance, Figure S8(a) displays a simulated diffraction pattern, and Figure S8(b) shows its closest reference pattern. While the diffraction spots in the high-k region of the reference pattern are circularly smeared due to the small number of basis vectors employed in the reconstruction, the major patterns in the low-k region, which are responsible for the basic shape of the model, agree with each other.

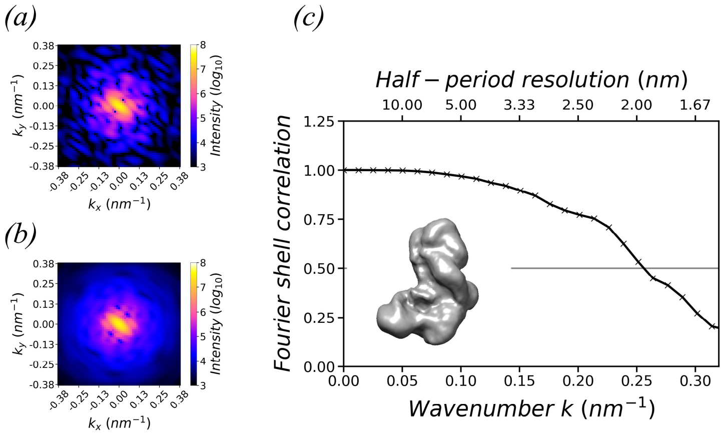

Figure S8 (a) is a noise-free simulated diffraction pattern and (b) is its closest reference pattern sliced from the reconstructed 3D diffraction intensity volume. (c) displays the updated reconstruction and its Fourier shell correlation with respect to the original model. The half-period resolution is 1.95 nm. The isosurface is plotted at 10% of the maximum density.

To validate the orientation estimation, we build an updated 3D diffraction intensity volume by positioning all 10,000 simulated diffraction patterns at their assigned orientations. The updated 3D diffraction intensity volume is expressed in Cartesian coordinates. Its value at a given grid point (x, y, z) is determined by taking the arithmetic mean of the values at all inserted points within the voxel centred at (x, y, z). This insertion method can be accepted when the inserted points are sufficiently dense. After phase retrieval, we obtain the updated reconstructed model and its FSC with respect to the original model, as show in Figure S8(c). The half-period resolution is 1.95 nm, which is higher than 4.07 nm resolution of the original reconstruction. This improvement confirms the effectiveness of the orientation estimation. With this approach, the angles can be estimated without iterative procedures commonly used in orientation-based approaches.

The method mentioned above can also be applied to noisy diffraction patterns. We test two datasets contributed to the reconstructions in Figure S9 (b) and (c). The incident laser fluxes are 10^{13} and 10^{12} photons per square micron per shot, respectively. Photon shot noise is added during the simulation. The updated reconstructed models have half-period resolutions of 3.04 nm and 3.06 nm, respectively, which are slightly larger than the voxel size of 2.5 nm and better than the original reconstructions' 4.07 nm and 3.99 nm.

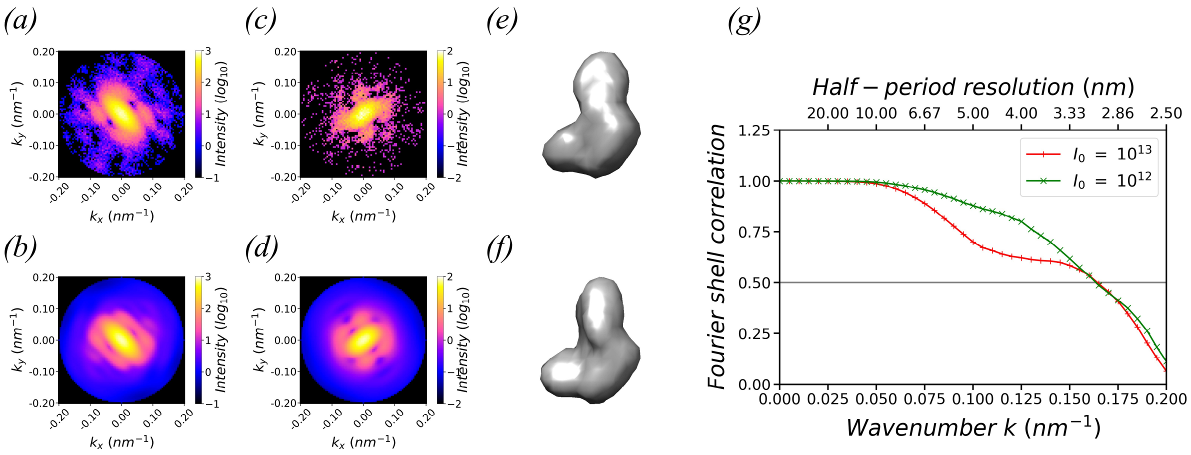

Figure S9 (a) and (c) are simulated noisy diffraction patterns when the incident laser fluxes are 10^{13} and 10^{12} photons per square micron per shot, respectively. (b) and (d) are their respective closest reference patterns sliced from the reconstructed 3D diffraction intensity volumes. (e) and (f) are the corresponding updated reconstructed models. The isosurfaces are plotted at 10% of the maximum density. (g) plots their Fourier shell correlations with respect to the original model. They have half-period resolutions of 3.04 nm and 3.06 nm, respectively.

In this illustration, the limited resolution of the correlation-based reconstruction is mainly attributed to the employment of only 21 basis vectors. The maximum degree l of these 21 basis vectors is only 14. As a result, even if they can be perfectly aligned, the reconstructed 3D diffraction intensity volume is smeared and the high-frequency information cannot be restored. In contrast, in the updated 3D diffraction intensity volume which is constructed by positioning all the original diffraction patterns at their assigned orientations, the high-frequency information is preserved, and consequently the resolution of reconstruction is improved.

S6. Verification of parameters α and β using experimental diffraction patterns

The parameters α and β in Eq. (A11) correspond to the detector's Fano noise and system electronic noise, respectively. In general, they are expected to be accurately known. On the other hand, they can also be estimated or verified using experimental diffraction patterns. For example, Figure S10 shows a part of the frequency histogram of photon counts in the dataset with the noise, which was used as input for the reconstruction in Figure 6(b). Clearly, peaks are present at integer photon counts. If the detector is an ideal photon-counting detector, the diffraction patterns are free of detector noise and these peaks should resemble delta functions. However, for a photon-integrating detector, affected by the Fano noise and the system

electronic noise, these peaks have the shape of Gaussian functions with certain widths. We use σ_N to denote the standard deviation of the peak at photon counts of N, then we have

$$\sigma_N^2 = N\alpha + \beta. \qquad (S4)$$

This equation enables the estimation or verification of the values of α and β.

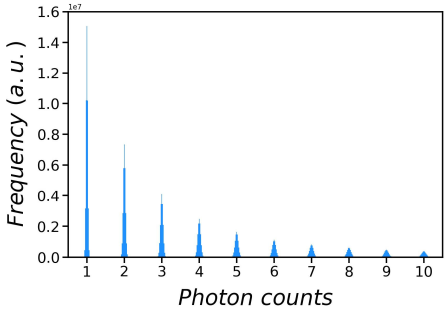

Figure S10 Frequency of photon counts in simulated noisy diffraction patterns.

In a real experiment, the values of α and β may fluctuate with changes in environmental conditions and the detector's state. Additionally, the peak's shape may not be Gaussian but rather pseudo-Voigt. Nevertheless, it still corresponds to the statistical frequency distribution of detector noise and allows for estimating the second moment of the noise. In some cases, the frequency histogram in the low-count region may have a continuous background resulting from the leak current from high-photon-count pixels. To address this issue, it is effective to count only the medium-k regions in the diffraction patterns where the number of high-photon-count pixels is small. Additionally, occasional charge sharing between adjacent pixels may smear the peaks in the frequency histogram. Since the pattern and the occurrence probability of charge sharing vary significantly under different detector conditions, here we will not discuss it in detail.
